# Optimizing wave energy converter benchmarking with a fuzzy-based decision-making approach

**DOI:** 10.1371/journal.pone.0307894

**Published:** 2024-07-26

**Authors:** Nhat-Luong Nhieu, Tri Dung Dang

**Affiliations:** College of Technology and Design, University of Economics Ho Chi Minh City, Ho Chi Minh City, Vietnam; Gonbad Kavous University, ISLAMIC REPUBLIC OF IRAN

## Abstract

The quest for sustainable energy solutions has intensified interest in marine renewables, particularly wave energy. This study addresses the crucial need for an objective assessment of Wave Energy Converter (WEC) technologies, which are instrumental in harnessing ocean waves for electricity generation. To benchmark WEC technologies, we employed an integrated approach combining the MEthod based on the Removal Effects of Criteria (MEREC) and the Spherical Fuzzy Combine Compromise Solution (SF-CoCoSo). MEREC provided a systematic way to determine the importance of various benchmarking criteria, while SF-CoCoSo facilitated the synthesis of complex decision-making data into a coherent evaluation score for each technology. The results of the study offer a definitive ranking of WEC technologies, with findings emphasizing the importance of grid connectivity and adaptability to various wave conditions as pivotal to the technologies’ success. While the study makes significant strides in the evaluation of WECs, it also recognizes limitations, including the potential for evolving market dynamics to influence criteria weightings and the assumption that the MCDM methods capture all decision-making complexities. Future work should expand the evaluative criteria and explore additional MCDM methods to validate and refine the benchmarking process further.

## 1. Introduction

The quest for sustainable and renewable energy sources has led to a growing interest in wave energy, recognized for its immense potential and significance [1]. Unlike other renewable sources, wave energy offers a consistent and powerful supply, largely untapped and capable of meeting global energy demands multiple times over [2]. Its exploitation promises a revolution in the energy sector, providing a clean, inexhaustible energy source that could significantly reduce our reliance on fossil fuels [3]. The environmental benefits of wave energy are notable as well, offering a greener alternative that minimizes carbon footprint and ecological disruption, making it a pivotal element in the transition towards sustainable energy solutions [4]. As wave energy technology evolves, the importance of benchmarking WEC technologies cannot be overstated. Benchmarking serves as a critical evaluative process to compare and contrast different WEC systems, aiming to identify the most effective and cost-efficient among them [5]. This process is vital for the continual improvement and innovation within the field of wave energy. It provides valuable insights for developers, investors, and policymakers, helping to shape future developments, allocate resources wisely, and establish industry standards [6]. Effective benchmarking can accelerate the adoption of wave energy by highlighting successful technologies and practices, thereby paving the way for wider acceptance and implementation.

In addressing the intricate task of benchmarking WEC technologies, Multiple Criteria Decision-Making (MCDM) methods emerge as powerful tools. These methods enable a holistic and nuanced analysis by considering a wide range of criteria, from technical performance and economic feasibility to environmental impact and social acceptance [7, 8]. MCDM facilitates balanced evaluation, accommodating the multifaceted nature of decision-making in this context. This approach is especially pertinent given the diverse and sometimes conflicting criteria involved in assessing wave energy technologies, ensuring that decisions are well-rounded and robust [9]. In widely used MCDM methods, the MEthod based on the Removal Effects of Criteria (MEREC) stands out for its objective approach to determining the weight of various criteria. It systematically analyzes the impact of removing a criterion, thereby revealing its relative importance in the overall decision-making process [10]. This method ensures that each criterion’s contribution is accurately reflected, leading to more balanced and equitable decision-making. On the other hand, the Spherical Fuzzy Combine Compromise Solution (SF-CoCoSo) method introduces an advanced level of decision analysis by incorporating spherical fuzzy sets. Unlike conventional fuzzy numbers, which typically model uncertainty using a single membership function, spherical fuzzy numbers extend this concept by incorporating three-dimensional membership, non-membership, and hesitancy degrees [11]. This richer representation allows SFNs to capture a more nuanced and accurate portrayal of uncertainty and vagueness inherent in human judgments. Traditional fuzzy sets and their extensions, such as intuitionistic and Pythagorean fuzzy sets, primarily focus on two dimensions, limiting their ability to fully encompass the complexities of decision-making scenarios. In contrast, SFNs offer enhanced flexibility and expressiveness, providing a comprehensive framework that improves the robustness and precision of MCDM analyses [12, 13]. This makes spherical fuzzy sets particularly useful in scenarios where decision data are highly uncertain and subject to multiple interpretations, thereby enhancing the overall reliability and effectiveness of the decision-making process [14, 15]. This approach allows for a more nuanced representation of uncertainty and vagueness inherent in human judgments [16]. SF-CoCoSo synthesizes these fuzzy evaluations into a comprehensive compromise solution, skillfully balancing between the best and most feasible options. The integration of MEREC and SF-CoCoSo in benchmarking WEC technologies promises a more refined, accurate, and comprehensive assessment, paving the way for identifying the most promising and efficient wave energy converters. This innovative combination marks a significant advancement in the field, offering robust tools for tackling the complexities of technology assessment in renewable energy systems.

Despite comprehensive insights from the literature on WECs and MCDM, a research gap exists in integrating advanced fuzzy logic with objective weighting methods for WEC benchmarking. Specifically, studies leveraging spherical fuzzy sets with the MEREC method are scarce. This presents an opportunity to improve objectivity and precision in WEC assessments by addressing uncertainty and subjectivity. Furthermore, while methods like CoCoSo balance competing criteria, their application in WEC benchmarking, especially with spherical fuzzy logic, remains underexplored. This study aims to fill these gaps by developing a fuzzy-based, objectively weighted decision-making approach, refining the methodology for sustainable energy decisions.

The motivation behind employing an integrated MCDM approach in this study stems from the recognition of the complex and multi-dimensional challenges inherent in benchmarking WEC technologies. By combining various MCDM methodologies, this approach seeks to address the diverse set of criteria involved in evaluating WEC technologies. This integration aims to refine the decision-making process, enhancing its accuracy, comprehensiveness, and reliability. It represents an innovative step forward in tackling the intricate task of benchmarking in the wave energy sector, potentially leading to more informed and effective decisions.

This study is primarily aimed at advancing the benchmarking process of WEC technologies through the integration of two distinct methodologies: the objective weighting capabilities of the MEREC and the nuanced decision analysis afforded by the SF-CoCoSo method. By fusing these approaches, the research endeavors to provide a comprehensive and balanced evaluation of WEC technologies. The pivotal role of benchmarking WEC technologies for advancing wave energy as a viable and sustainable energy source is underscored. It not only identifies leading technologies but also informs policy, guides research and development efforts, and encourages industry-wide standards and best practices [6].

An innovative, integrated MCDM approach to the benchmarking of WEC technologies is contributed by this study, promising to enhance the clarity, accuracy, and effectiveness of technology assessments. Through this pioneering methodology, the strategic development and deployment of wave energy converters are aimed to be supported, marking a crucial step forward in the sustainable harnessing of wave energy.

## 2. Literature review

### 2.1. WECs technology studies

The literature on WECs is extensive, reflecting the diversity of designs and approaches to harnessing wave power. Fundamental to the body of research is the principle that WECs must be efficient, durable, and environmentally sustainable to be viable long-term solutions. Early studies trace the historical development of WECs, noting initial concepts dating back to the 1970s. Over the years, various designs have been proposed, such as point absorbers (oscillating bodies), attenuators, and oscillating water columns, each suited to different marine environments and wave conditions. Comparative analyses, such as those by Harris et al. (2004) or Folley and Whittaker (2010), provide a comprehensive overview of these technologies, discussing their operating principles, energy conversion mechanisms, and typical locations [17, 18]. A significant portion of the literature focuses on performance metrics for WECs. Researchers have established various efficiency indicators, including capture width ratio and power matrix, as benchmarks. Studies by Aderinto & Li (2019) and Majidi et al. (2021) have been instrumental in defining these metrics, which are crucial for understanding and improving WEC technology [19, 20]. The environmental impact of WECs is a critical aspect explored in the literature. Many studies examine the ecological effects of WEC installations, including potential impacts on marine life and habitats [21, 22]. Economically, the viability of WECs is often analyzed through cost-benefit analyses, levelized cost of energy (LCOE), and market potential assessments. Notable contributions by Chang et al. evaluate the economic challenges and opportunities for WEC technologies [23]. Studies concerning the technology readiness level (TRL) of WECs highlight the maturity of different WEC designs and their readiness for commercial deployment. Bertram et al. provide a TRL framework specific to WECs, while Magagna and Uihlein discuss the roadmaps and strategic actions required to advance WEC technologies to higher TRLs in Europe [24, 25]. The literature also delves into the challenges faced by WECs, such as those related to maintenance, scalability, and grid integration [1]. More recent literature reflects the ongoing innovations in WEC technology. Studies on new materials, advanced control systems, and optimization algorithms are frequent, with researchers exploring novel approaches to improve efficiency and resilience [2, 26]. The advent of digital twin technology and its application to WECs, as examined by Katsidoniotaki et al., exemplifies the technological advancements in this field [27]. Benchmarking studies, essential for comparative analysis and policy-making, have become increasingly prevalent. The application of MCDM methods to WEC technology assessment is a relatively recent trend in the literature, addressing the complexity of evaluating multiple performance criteria [7, 24, 28].

The literature on WEC technologies presents a multidimensional view of the field, covering a range of topics from foundational concepts to cutting-edge innovations. The collective research underscores the potential of WECs as a sustainable energy source while acknowledging the technical, environmental, and economic challenges that must be addressed. The continuous evolution of benchmarking methodologies, including MCDM approaches, reflects the dynamic nature of the field and the ongoing effort to optimize WEC technologies for global energy portfolios.

### 2.2. Studies of MCDM approaches

One of the critical steps in MCDM is the determination of the weights of criteria, which can significantly influence the final decision. The literature distinguishes between two main weight assignment methods: subjective and objective. Subjective methods rely on the judgment and preferences of the decision-maker. Techniques such as the Analytic Hierarchy Process (AHP) and Delphi method are widely discussed in the literature for their ability to capture expert opinions and preferences [29–31]. These methods, however, may introduce bias and are dependent on the expertise and consistency of the judgments provided. In contrast, objective methods determine weights based on the inherent data structure of the decision matrix, without relying on external judgments [32]. Techniques like Entropy and the CRiteria Importance Through Intercriteria Correlation (CRITIC) methods are frequently cited for their ability to reduce subjectivity by extracting weights from the variation in the criteria data [32–34]. The MEthod based on the MEREC is an objective weighting method that assesses the importance of criteria based on the sensitivity of the decision-making process to the removal of each criterion [10]. MEREC stands out in the literature for its unique approach to understanding the interdependencies and impact of each criterion on the overall decision-making process.

Compromise solution-based methods aim to find a solution that is the closest to the ideal and furthest from the anti-ideal solution. These methods are based on the concept of satisfying decision-making, where the goal is not to maximize or minimize individual criteria but to find a solution that is acceptable across all criteria [35]. The literature on compromise solution-based methods is rich with studies on the VIKOR method, which introduces the idea of ranking and selecting solutions based on their proximity to the ideal solution [36]. These methods are praised for their ability to provide a balance between different criteria, making them suitable for scenarios with competing and non-commensurable criteria. The Combined Compromise Solution (CoCoSo) method is a relatively recent addition to compromise solution-based MCDM methods. It combines the results of three different compromise ranking methods to derive a comprehensive solution [37]. The CoCoSo has gained attention for its robustness and the ability to produce a more stable and reliable ranking by mitigating the weaknesses of individual compromise methods [38].

Fuzzy extensions of traditional MCDM methods, like fuzzy AHP and fuzzy TOPSIS, have been extensively studied and applied across various fields. They are known for their ability to model the uncertainty of subjective assessments and to provide a more nuanced approach to decision-making [39]. Spherical fuzzy sets (SFS), an extension of fuzzy sets, offer a three-dimensional representation of membership, non-membership, and hesitancy degrees, providing an even more refined modeling of uncertainty [13]. The exploration of SFS in decision-making processes has gained significant momentum in recent research, emphasizing its effectiveness in handling uncertainty and vagueness across various domains. Kaushik D. and Sankar K.R. (2023) ventured into the T-spherical fuzzy set (T-SFS) domain, developing a hybrid form of operators to address biasness and ensure unbiased decision-making in MADM problems [40]. Their work highlighted the advantages of using weighted power partitioned neutral average and geometric operators within the T-SFS environment, showcasing its application in hydrogen refueling station site selection. Muhammad Saad and Ayesha Rafiq (2023) further expanded the utility of T-SFS by introducing correlation coefficients for T-SFS, demonstrating their application in pattern recognition and decision-making, notably in selecting a suitable COVID-19 mask [41]. Arun Sarkar et al. (2023) introduced an innovative model, the T-spherical fuzzy hypersoft set (T-SFHSS), enhancing the precision of fuzzy set calculations and proposing novel aggregation operators for T-SFHSS [42]. Their research underscored the model’s superiority in handling imprecise data, illustrated through an application in natural agribusiness. D. Ajay et al (2023) contributed by defining new exponential and Einstein exponential operational laws for SFS, aiming to refine decision-making processes in evaluating psychotherapy methods [43]. The integration of spherical fuzzy sets into MCDM methods allows for a comprehensive and sophisticated handling of uncertainty, enhancing the decision-making process’s flexibility and expressiveness [12, 16, 38, 44].

The literature on MCDM approaches reflects a continued evolution from traditional, more subjective methods to sophisticated, data-driven techniques that seek to reduce bias and better handle uncertainty. Objective methods like MEREC, compromise solution methods like CoCoSo, and the application of fuzzy theory, particularly spherical fuzzy sets, represent significant advancements in the field, offering nuanced and robust frameworks for decision-making in complex, multi-criteria environments.

### 2.3. MCDM Applications for WECs

The application of MCDM approaches in evaluating WECs represents a critical advancement in optimizing renewable energy technologies. MCDM methods facilitate comprehensive analyses by incorporating various technical, economic, social, and environmental criteria, essential for assessing the viability and sustainability of WEC technologies. Recent literature underscores the increasing reliance on MCDM methodologies for WEC assessments. Sadaf Nasrollahi et al. (2023) employed the fuzzy Delphi method and PROMETHEE to select optimal WEC technologies for the Caspian Sea, indicating the preference for Pelamis based on an extensive set of criteria [7]. Shadmani et al. (2023) developed a novel MCDM strategy integrating exploitable wave energy storage and production metrics to select optimal sites for WEC deployment along Oman’s coast [28]. Meng Shao et al. (2024) combined GIS, MCDM, and ANN techniques to enhance site selection and wave power forecasting for WPPs, focusing on Hainan Island and identifying suitable areas for deployment [45]. Daekook Kang et al. (2024) introduced an innovative hybrid MCDM methodology using fuzzy SWARA and ELECTRE for selecting the most suitable WEC, highlighting the point absorber technology [46]. Shabnam et al. (2023) emphasized the importance of combining offshore wind and wave energy through MCDM methods to identify the best locations for constructing combined farms, yet noted the lack of evaluation on seabed conditions and climate change impacts [47].

The findings from these studies reflect the effectiveness of MCDM approaches in navigating the complexities associated with WEC technology assessment and site selection. These methodologies offer a structured framework for integrating diverse criteria, ensuring a holistic evaluation of potential technologies and locations. The adoption of fuzzy logic and other advanced techniques further enhances the decision-making process, accommodating uncertainty and subjectivity in assessments. Despite the progress in applying MCDM to WEC assessments, a notable gap remains in fully exploiting the potential of fuzzy-based objective weighting methods, particularly in integrating spherical fuzzy sets with the MEREC method. Most studies focus on traditional MCDM approaches without fully embracing the advancements in fuzzy logic to handle uncertainty and hesitancy more effectively.

While the existing literature on WECs and Multi-Criteria Decision-Making MCDM approaches offers comprehensive insights into the technical, environmental, and economic aspects of WEC technologies, as well as various methodologies for their evaluation, a noticeable research gap persists in the integration of advanced fuzzy logic with objective weighting methods for the benchmarking of WEC technologies. Specifically, there is a scarcity of studies that leverage the nuanced capabilities of spherical fuzzy sets in conjunction with the MEREC method to refine the weighting and evaluation process. This gap indicates an opportunity for a novel approach that enhances the objectivity and precision of WEC technology assessment by accounting for the inherent uncertainty and subjectivity in decision-making processes. Additionally, while compromise solution-based methods like CoCoSo have been recognized for their robustness in balancing competing criteria, their application in the context of WEC technology benchmarking, particularly when integrated with spherical fuzzy logic, remains underexplored. This study aims to bridge these gaps by developing and applying a fuzzy-based, objectively weighted integrated decision-making approach, thereby contributing a refined methodology for benchmarking WEC technologies that better reflects the complex, multidimensional nature of sustainable energy decisions.

## 3. Methodology

### 3.1. Preliminary

**Fig 1:**
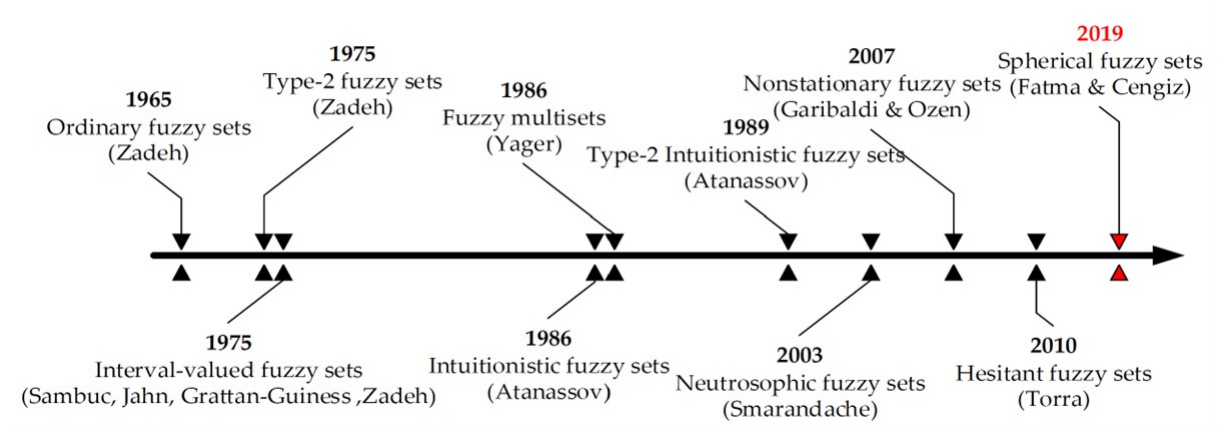
Development of fuzzy sets [16].

Fuzzy theory, introduced by Lotfi Zadeh in the 1960s, is a mathematical framework for dealing with uncertainty and imprecision, challenging the traditional binary logic of true or false by introducing degrees of truth [48]. This theory allows for more nuanced and realistic modeling of complex systems where clear-cut boundaries do not exist [49]. Building on this concept, SFS, a more recent development in fuzzy logic, offers an advanced approach to handling uncertainty as shown in Fig 1 [13]. It extends the traditional fuzzy set and intuitionistic fuzzy set by incorporating a third parameter, which enhances its ability to represent and process vagueness and ambiguity in data [50]. This three-dimensional representation in spherical fuzzy sets offers a more comprehensive and flexible tool for dealing with uncertain information, making it valuable in fields like artificial intelligence, decision-making, and complex system analysis [51].

**Definition 1.** In the universe of discourse S, the spherical fuzzy set *Ñ* is defined by

$$\tilde{N}=\left\{\langle \left.s,\left(\vartheta_{\tilde{N}}\left(s\right),\mu_{\tilde{N}}\left(s\right),\pi_{\tilde{N}}\left(s\right)\right)\right|s\in S\right\}$$ (1)

where

$$\vartheta_{\tilde{N}},\;\mu_{\tilde{N}},\;\pi_{\tilde{N}}:S\to \left[0,\;1\right]\;\;and\;0\leq \vartheta_{\tilde{N}}^{2}\left(x\right)+\mu_{\tilde{N}}^{2}\left(x\right)+\pi_{\tilde{N}}^{2}\left(x\right)\leq 1\;\forall s\in S$$ (2)

The parameters *ϑ*_*Ñ*_ (*s*), *μ*_*Ñ*_ (*s*), and *π*_*Ñ*_ (*s*) are the membership degree, non-membership degree, and hesitancy degree of each *s* to *Ñ*, respectively.

**Definition 2.** In the universe of discourse *S*_1_ and *S*_2_, two spherical fuzzy numbers (SFN) *Ñ* = (*ϑ*_*Ñ*_, *μ*_*Ñ*_, *π*_*Ñ*_) and $\tilde{M}=(\vartheta_{\tilde{M}},\mu_{\tilde{M}},\;\pi_{\tilde{M}})$ have the basic operators are defined as Eqs (3)–(5):

$$\tilde{N}⊕\tilde{M}=\left\{{\left(\vartheta_{\tilde{N}}^{2}+\vartheta_{\tilde{M}}^{2}-\vartheta_{\tilde{N}}^{2}\vartheta_{\tilde{M}}^{2}\right)}^{\frac{1}{2}},\;\mu_{\tilde{N}}\mu_{\tilde{M}},\;{\left(\left(1-\vartheta_{\tilde{M}}^{2}\right)\pi_{\tilde{N}}^{2}+\left(1-\vartheta_{\tilde{N}}^{2}\right)\pi_{\tilde{M}}^{2}-\pi_{\tilde{N}}^{2}\pi_{\tilde{M}}^{2}\right)}^{\frac{1}{2}}\right\}$$ (3)

$$\tilde{N}⊗\tilde{M}=\left\{\vartheta_{\tilde{N}}\vartheta_{\tilde{M}},\;{\left(\mu_{\tilde{N}}^{2}+\mu_{\tilde{M}}^{2}-\mu_{\tilde{N}}^{2}\mu_{\tilde{M}}^{2}\right)}^{\frac{1}{2}},\;{\left(\left(1-\mu_{\tilde{M}}^{2}\right)\pi_{\tilde{N}}^{2}+\left(1-\mu_{\tilde{N}}^{2}\right)\pi_{\tilde{M}}^{2}-\pi_{\tilde{N}}^{2}\pi_{\tilde{M}}^{2}\right)}^{\frac{1}{2}}\right\}$$ (4)

$$\alpha \tilde{N}=\left\{{\left(1-{\left(1-\vartheta_{\tilde{N}}^{2}\right)}^{\alpha }\right)}^{\frac{1}{2}},\;\mu_{\tilde{N}}^{\alpha },\;{\left({\left(1-\vartheta_{\tilde{N}}^{2}\right)}^{\alpha }-{\left(1-\vartheta_{\tilde{N}}^{2}-\pi_{\tilde{N}}^{2}\right)}^{\alpha }\right)}^{\frac{1}{2}}\right\},\;\forall \alpha >0$$ (5)

$${\tilde{A}}^{\alpha }=\left\{\vartheta_{\tilde{N}}^{\alpha },{\left(1-{\left(1-\mu_{\tilde{N}}^{2}\right)}^{\alpha }\right)}^{\frac{1}{2}},{\left({\left(1-\mu_{\tilde{N}}^{2}\right)}^{\alpha }-{\left(1-\mu_{\tilde{N}}^{2}-\pi_{\tilde{N}}^{2}\right)}^{\alpha }\right)}^{\frac{1}{2}}\right\},\;\forall \alpha >0$$ (6)

**Definition 3.** Consider the weight vector *φ* = (*φ*_1_, *φ*_2_, …, *φ*_*m*_), where 0 ≤ *φ*_*i*_ ≤ 1 and $\sum_{i=1}^{m}\varphi_{i}=1$. Spherical weighted arithmetic mean (SWAM) and spherical weighted geometric mean (SWGM) are defined as Eqs (7) and (8):

$$\begin{array}{c} SWAM_{\varphi }\left({\tilde{N}}_{1},\;{\tilde{N}}_{2},\ldots ,\;{\tilde{N}}_{m}\right)=\varphi_{1}{\tilde{N}}_{1}+\varphi_{2}{\tilde{N}}_{2}+\cdots +\varphi_{m}{\tilde{N}}_{m}\\ =\;\left\{{\left(1-\overset{m}{\underset{i=1}{\prod }}{\left(1-\vartheta_{{\tilde{N}}_{i}}^{2}\right)}^{\varphi_{i}}\right)}^{\frac{1}{2}},\overset{m}{\underset{i=1}{\prod }}\mu_{{\tilde{N}}_{i}}^{\varphi_{i}},{\left(\overset{m}{\underset{i=1}{\prod }}{\left(1-\vartheta_{{\tilde{N}}_{i}}^{2}\right)}^{\varphi_{i}}-\overset{m}{\underset{i=1}{\prod }}{\left(1-\vartheta_{{\tilde{N}}_{i}}^{2}-\pi_{{\tilde{N}}_{i}}^{2}\right)}^{\varphi_{i}}\right)}^{\frac{1}{2}}\right\} \end{array}$$ (7)

$$\begin{array}{c} SWGM_{\varphi }\left({\tilde{N}}_{1},\;{\tilde{N}}_{2},\ldots ,\;{\tilde{N}}_{m}\right)={\tilde{N}}_{1}^{\varphi_{1}}+{\tilde{N}}_{2}^{\varphi_{2}}+\cdots +{\tilde{N}}_{m}^{\varphi_{m}}\\ =\left\{\overset{m}{\underset{i=1}{\prod }}\vartheta_{{\tilde{N}}_{i}}^{\varphi_{i}},{\left(1-\overset{m}{\underset{i=1}{\prod }}{\left(1-\mu_{{\tilde{N}}_{i}}^{2}\right)}^{\varphi_{i}}\right)}^{\frac{1}{2}},{\left(\overset{m}{\underset{i=1}{\prod }}{\left(1-\mu_{{\tilde{N}}_{i}}^{2}\right)}^{\varphi_{i}}-\overset{m}{\underset{i=1}{\prod }}{\left(1-\mu_{{\tilde{N}}_{i}}^{2}-\pi_{{\tilde{N}}_{i}}^{2}\right)}^{\varphi_{i}}\right)}^{\frac{1}{2}}\right\} \end{array}$$ (8)

**Definition 4.** Let *Ñ* = (*ϑ*_*Ñ*_, *μ*_*Ñ*_, *π*_*Ñ*_) and $\tilde{M}=(\vartheta_{\tilde{M}},\mu_{\tilde{M}},\;\pi_{\tilde{M}})$ be two spherical fuzzy numbers from the universe of discourse *S*_*1*_ and *S*_2_. The followings are valid under the condition *α*, *α*_1_, *α*_2_ > 0 [13].


$$\tilde{N}⊕\tilde{M}=\tilde{M}⊕\tilde{N}$$ (9)

$$\tilde{N}⊕\tilde{M}=\tilde{M}⊕\tilde{N}$$ (10)

$$\alpha \left(\tilde{N}⊕\tilde{M}\right)=\alpha \tilde{N}⊕\alpha \tilde{M}$$ (11)

$$\alpha_{1}\tilde{N}⊕\alpha_{2}\tilde{N}=(\alpha_{1}+\alpha_{2})\tilde{N}$$ (12)

$${\left(\tilde{N}⊗\tilde{M}\right)}^{\alpha }={\tilde{N}}^{\alpha }⊗{\tilde{M}}^{\alpha }$$ (13)

$${\tilde{N}}^{\alpha_{1}}⊗{\tilde{N}}^{\alpha_{2}}={\tilde{N}}^{\alpha_{1}+\alpha_{2}}$$ (14)

**Definition 5.** The defuzzied value (DV) of spherical fuzzy number *Ñ* = (*ϑ*_*Ñ*_, *μ*_*Ñ*_, *π*_*Ñ*_) is defined as Eq (15) [52]:

$$DV\left(\tilde{N}\right)=\sqrt{100\times \left|{\left(3\times \vartheta_{\tilde{N}}-\frac{\pi_{\tilde{N}}}{2}\right)}^{2}-{\left(\frac{\mu_{\tilde{N}}}{2}-\pi_{\tilde{N}}\right)}^{2}\right|}$$ (15)

### 3.2. The proposed spherical fuzzy objectively weighting integrated decision-making approach

To take advantage of the advantages of MEREC and SF-CoCoSo, this study introduces an integrated approach which is performed according to the following procedure:

**Step 1.** Experts (*k* = 1 … *K*) (or decision makers), who have expertise and experience in the field, are identified. Based on their expertise, the weights of the experts are determined. With the given SFN ${\tilde{E}}^{k}=\left(\vartheta_{{\tilde{E}}^{k}},\mu_{{\tilde{E}}^{k}},\pi_{{\tilde{E}}^{k}}\right)$ representing the expertise of the *k*th expert, the weight (*ω*_*k*_) of the *k*th expert is determined as Eq (16) [53].

$$\omega_{k}=\frac{1-{\left(\left(1-\vartheta_{{\tilde{E}}^{k}}^{2}\right)+\mu_{{\tilde{E}}^{k}}^{2}+\pi_{{\tilde{E}}^{k}}^{2})/3\right)}^{\frac{1}{2}}}{\sum_{l=1}^{h}\left(1-{\left(\left(1-\vartheta_{{\tilde{E}}^{l}}^{2}\right)+\mu_{{\tilde{E}}^{l}}^{2}+\pi_{{\tilde{E}}^{l}}^{2})/3\right)}^{\frac{1}{2}}\right)}$$ (16)

where

$$\sum_{k=1}^{K}\omega_{k}=1\;\text{and}\;0\leq \;\vartheta_{{\tilde{E}}^{k}}^{2}+\mu_{{\tilde{E}}^{k}}^{2}+\pi_{{\tilde{E}}^{k}}^{2}\leq 1$$

As described in Table 1, SFN *Ẽ*_*k*_ representing the expertise of experts provided by analysts or higher-level decision-makers in linguistic terms based on expert attributes such as years of experience, qualifications.

**Table 1:** Linguistic terms and corresponding SFN for experts’ expertise.

Linguistic term	Spherical fuzzy number (*ϑ*, *μ*, *π*)
Very high (VH)	(0.85, 0.15, 0.45)
High (H)	(0.60, 0.20, 0.35)
Moderate (M)	(0.35, 0.25, 0.25)

**Step 2:** The benchmarking criteria (*j* = 1 … *J*), and alternatives (*i* = 1 … *I*) are defined based on literature review and experts’ opinions.

**Step 3.** Experts provide linguistic assessments of alternatives according to the criteria. These linguistic assessments are then transformed into the corresponding SFNs as shown in Table 2, which is provided by experts or decision makers, to form SF decision matrices. SF decision matrices are represented as Eq (17). In other applications, the linguistic scale can be defined by decision makers or experts. The SFN values should be symmetrically distributed around the neutral point, designated as "Medium" (0.500, 0.500, 0.500), ensuring a balanced and consistent progression in the evaluation scale. This symmetry implies that as judgments move from neutral to extremely positive or negative, the membership and non-membership degrees adjust inversely, maintaining logical coherence [11, 15, 16, 31].


$${\tilde{N}}^{k}={\left[{\tilde{n}}_{ij}^{k}\right]}_{I\times J}$$ (17)

**Table 2:** Linguistic terms and corresponding SFN for alternative assessments.

Linguistic term	Spherical fuzzy number (*ϑ*_*Ñ*_, *μ*_*Ñ*_, *π*_*Ñ*_)
Low (L)	(0.040, 0.960, 0.040)
Slightly low (SL)	(0.270, 0.730, 0.270)
Moderate (M)	(0.500, 0.500, 0.500)
Slightly high (SH)	(0.730, 0.270, 0.270)
High (H)	(0.960, 0.040, 0.040)

**Step 4.** The aggregated decision matrix is constructed using the SWAM based on experts’ weights as Eqs (18) and (19).

$$\tilde{N}={\left[{\tilde{n}}_{ij}\right]}_{I\times J}$$ (18)

where

$${\tilde{n}}_{il}=SWAM_{\omega_{k}}\left({\tilde{n}}_{il}^{1},{\tilde{n}}_{il}^{2},\ldots ,{\tilde{n}}_{il}^{K}\right)=\omega_{1}{\tilde{n}}_{il}^{1}+\omega_{2}{\tilde{n}}_{il}^{1}+\cdots +\omega_{K}{\tilde{n}}_{il}^{K},\;i=1\ldots I,\;l=1\ldots L$$ (19)

**Step 5.** The crisp decision matrix is constructed according to Eq (15).


$$N={\left[n_{ij}\right]}_{I\times J}$$ (20)

**Step 6.** The crisp decision matrix is normalized, according to Eqs (21) and (22), to transform all criteria into the non-beneficial criteria.

$$m={\left[m_{ij}\right]}_{I\times J}$$ (21)

where

$$m_{ij}=\frac{{\text{min}}_{i}\;n_{ij}}{n_{ij}}$$ (22)

**Fig 2:**
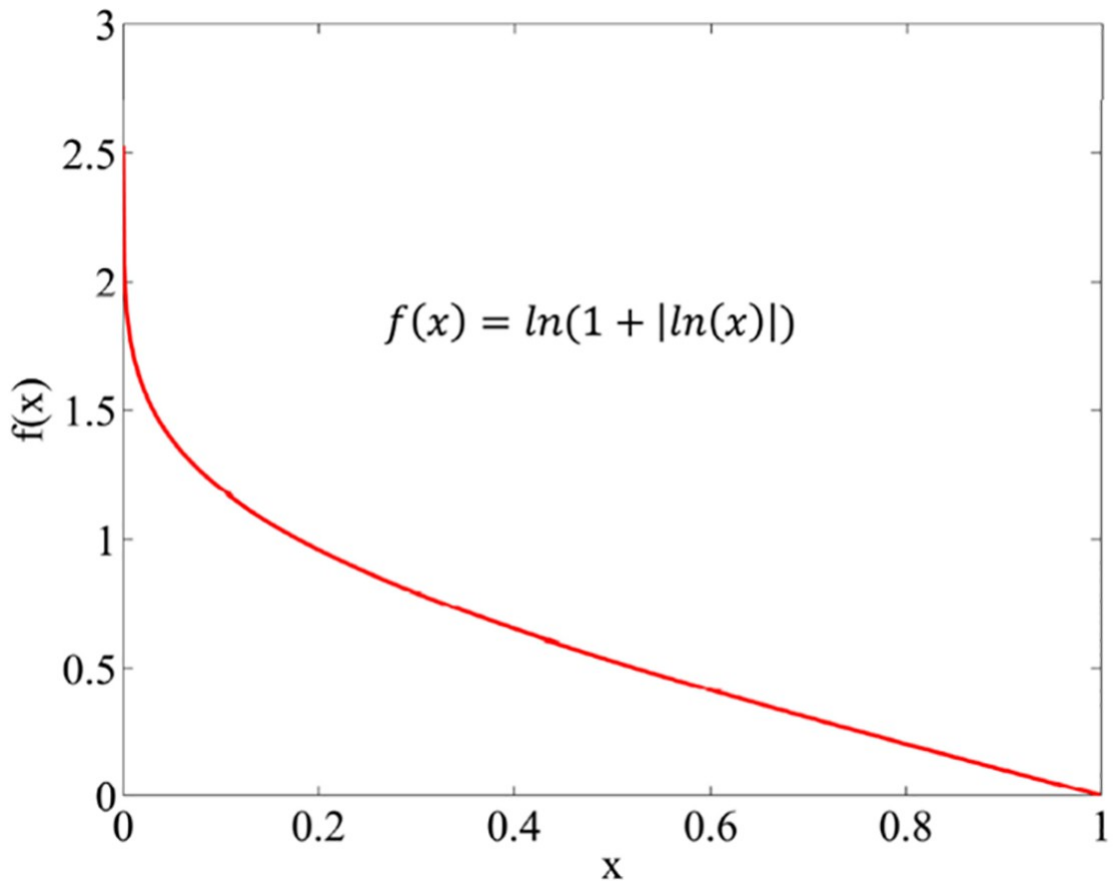
The weights of the comparative analysis.

**Step 7.** The overall performance of the alternatives (*S*_*i*_) is calculated using a logarithmic measure with equal criteria weights according to Eq (23). It is based on the non-linear function as shown in Fig 2. Therefore, the smaller value of *m*_*ij*_ yield higher value of *S*_*i*_.


$$S_{i}=\text{ln}\left(1+\left(\frac{1}{J}\overset{J}{\underset{j=1}{\sum }}\left|\text{ln}\left(m_{ij}\right)\right|\right)\right)$$ (23)

**Step 8.** The removal overall performance of the alternatives $\left(S_{ij}^{\prime }\right)$ calculated as the Step 7 but one-by-one criterion is removed from the matrix as shown on Eq (24). The removal overall performance of the alternatives $\left(S_{ij}^{\prime }\right)$ is denoted for the overall performance of *ith* alternatives with the removal of *jth* criterion.


$$S_{ij}^{\prime }=\text{ln}\left(1+\left(\frac{1}{J}\overset{J}{\underset{k=1,k\neq j}{\sum }}\left|\text{ln}\left(m_{ik}\right)\right|\right)\right)$$ (24)

**Step 9.** The weights of criteria (*w*_*j*_) are computed based on the removal effect (*E*_*j*_) of the *jth* criterion according to Eq (25).


$$w_{j}=\frac{E_{j}}{\sum_{j=1}^{J}E_{j}}\;where\;E_{j}=\overset{I}{\underset{i=1}{\sum }}\left|S_{ij}^{\prime }-S_{i}\right|$$ (25)

**Step 10.** Based on the criteria weight (*w*_*l*_), the weighted sequences of alternatives are determined using the SWAM and the SWGM according to Eqs (26) and (27). They are denoted as ${\tilde{SWA}}_{i}$ and ${\tilde{SWG}}_{i}$, respectively.


$${\tilde{SWA}}_{i}=w_{1}{\tilde{n}}_{i1}+w_{2}{\tilde{n}}_{i2}+\cdots +w_{j}{\tilde{n}}_{ij}+\cdots +w_{J}{\tilde{n}}_{iJ},\;i=1\ldots I$$ (26)

$${\tilde{SWG}}_{i}={\tilde{n}}_{i1}^{w_{1}}+{\tilde{n}}_{i2}^{w_{2}}+\cdots +{\tilde{n}}_{ij}^{w_{j}}+\cdots +{\tilde{n}}_{iJ}^{w_{J}},\;i=1\ldots I$$ (27)

**Step 11.** The defuzzied value of ${\tilde{SWA}}_{i}$ and ${\tilde{SWG}}_{i}$ are determined and denoted as *SWA*_*i*_ and *SWG*_*i*_ according to Eq (15).

**Step 12.** The additive normalized importance ($\Phi_{i}^{a}$) and the relative importance ($\Phi_{i}^{b})$ of the SWAM and the SWGM are calculated as Eqs (28) and (29), respectively. In addition, the trade-off importance ($\Phi_{i}^{c})$ of the alternatives is determined as Eq (30) with the stability and flexibility represented by the coefficient *δ*, which is selected by the decision-makers.


$$\Phi_{i}^{a}=\frac{SWA_{i}+SWG_{i}}{\sum_{i=1}^{I}(SWA_{i}+SWG_{i})},\;i=1\ldots I$$ (28)

$$\Phi_{i}^{b}=\frac{SWA_{i}}{\underset{i}{\text{min}}(SWA_{i})}+\frac{SWG_{i}}{\underset{i}{\text{min}}(SWG_{i})},\;i=1\ldots I$$ (29)

$$\Phi_{i}^{c}=\frac{\delta SWA_{i}+\left(1-\delta \right)SWG_{i}}{\delta \underset{i}{\text{min}}(SWA_{i})+\left(1-\delta \right)\underset{i}{\text{max}}\left(SWG_{i}\right)},\;i=1\ldots I$$ (30)

**Step 13.** The final evaluation score (Φ_*i*_) of alternatives is determined as Eq (31). The final rank of alternatives is ranked in descending order of the value of Φ_*i*_. In other words, the best alternative has the largest value of Φ_*i*_.


$$\Phi_{i}=\frac{\left(\Phi_{i}^{a}+\Phi_{i}^{b}+\Phi_{i}^{c}\right)}{3}+\sqrt[{3}]{\left(\Phi_{i}^{a}\times \Phi_{i}^{b}\times \Phi_{i}^{c}\right)},\;i=1\ldots I$$ (31)

## 4. Numerical results

### 4.1. WECs benchmarking by the proposed approach

Based on the references, the WEC technologies that can be considered during benchmarking include the Oscillating water columns (OWC), Point absorbers (PAB), Attenuators (ATE), and Overtopping devices (OTD). Moreover, we add two more Ocean Energy Systems which are Salinity gradient power (SGP) and Tidal stream turbines (TST) to the benchmarking to be evaluated [44]. Oscillating water columns (OWCs) utilize the movement of water in a chamber to drive an air turbine for electricity generation, making them a straightforward and efficient choice, primarily suited for regions with robust wave action. Point absorbers or oscillating bodies, anchored to the seabed, translate their motion with the waves into electricity through hydraulic or mechanical systems, offering versatility across different wave conditions. Attenuators, designed to absorb wave energy and dissipate it as heat or sound, serve the dual purpose of coastline protection and electricity generation. Salinity gradient power (SGP) devices, though still in early development stages, exploit salinity differences between seawater and freshwater as a potential cost-effective source of wave energy. Tidal stream turbines, resembling wind turbines but designed for tidal currents, are adaptable for both shallow and deep waters, representing a mature technology. Lastly, overtopping devices collect water from wave overtopping barriers to drive turbines or pumps efficiently, suitable for regions with high wave heights.

To perform benchmarking of WEC technologies, a group of six experts was first convened to conduct a survey using the Delphi method. Based on their expertise, as shown in Table 3, the corresponding SFN is recommended according to Table 1. After that, the experts’ weights are calculated according to Eq (16) and shown in Table 3. In the next step, through the Delphi method interview process, experts propose benchmarking criteria (BC) as well as provide linguistics judgments for WECs technologies corresponding to each BC. Table 4 below presents the BAs and linguistics judgments of the first expert. Based on the corresponding SFNs in Table 2, each expert’s linguistic judgments were converted into SFNs. The result of this process is the formation of individual SF benchmarking matrices. Based on the weights of the experts, which were obtained above, the individual benchmarking matrices are aggregated according to Eqs (18) and (19). The aggregated SF benchmarking matrix is shown in Table 5. To start the procedure to determine the objective weights of the BCs, the defuzzification and normalization process is performed according to Eqs (15) and (22), respectively. As the results, the obtained the crisp benchmarking matrix and the normalized benchmarking matrix are presented in Table A1 and Table A2 in S1 Appendix. As described in Eqs (23)–(25), the removal effects of the benchmarking criteria are calculated as shown in Table 6.

**Table 3:** Experts’ qualifications and weights.

Expert	Year of experience	Working/Research field	Highest degree	Recommended expertise	Corresponding SFN	Weight
1	12	Wave energy research	Ph.D.	Very high	(0.85, 0.15, 0.45)	0.178
2	13	Renewable energy manufacturer	Ph.D.	Very high	(0.85, 0.15, 0.45)	0.178
3	10	Renewable energy manufacturing	Master	High	(0.60, 0.20, 0.35)	0.167
4	8	Wave energy research	Ph.D.	High	(0.60, 0.20, 0.35)	0.167
5	11	Renewable energy investment	Master	High	(0.60, 0.20, 0.35)	0.167
6	5	Wave energy research	Master	Medium	(0.35, 0.25, 0.25)	0.143

**Table 4:** Benchmarking criteria and linguistics judgments by the 1^st^ expert.

Benchmarking criteria	Ref	WEC technology					
		OWC	PAB	ATE	SGP	TST	OTD
Efficiency (BC1)	[19, 54, 55]	L	SL	SL	SH	SL	L
Cost (BC2)	[19, 55–58]	SL	SH	SL	L	SH	SH
Robustness (BC3)	[54, 55, 57, 58]	L	SL	SH	M	SL	L
Environmental impact (BC4)	[55, 59]	SL	H	SH	SH	H	SH
Technology readiness level (BC5)	[54–58]	L	SL	SH	H	SL	SH
Required wave conditions (BC6)	[54, 55]	H	H	SH	SL	SL	SH
Power output (BC7)	[19, 54, 55]	SH	L	L	H	SH	SH
Operating range (BC8)	[54, 55]	L	SH	SH	H	SH	H
Survivability (BC9)	[55, 59]	H	SH	M	L	M	SL
Ease of installation and maintenance (BC10)	[54–56]	SH	H	SL	SL	M	L
Grid connection (BC11)	[54, 55]	H	M	M	H	SL	L
Social acceptance (BC12)	[55, 59]	L	H	M	M	SH	SH

**Table 5:** The aggregated SF benchmarking matrix.

BC	OWC	PAB	ATE	SGP	TST	OTD
BC1	(0.750, 0.284, 0.257)	(0.473, 0.605, 0.256)	(0.578, 0.518, 0.265)	(0.435, 0.625, 0.319)	(0.378, 0.714, 0.240)	(0.768, 0.306, 0.240)
BC2	(0.661, 0.422, 0.266)	(0.751, 0.287, 0.258)	(0.736, 0.291, 0.323)	(0.780, 0.273, 0.244)	(0.623, 0.395, 0.353)	(0.462, 0.591, 0.373)
BC3	(0.843, 0.192, 0.233)	(0.767, 0.308, 0.246)	(0.692, 0.367, 0.302)	(0.503, 0.565, 0.319)	(0.534, 0.509, 0.322)	(0.803, 0.238, 0.269)
BC4	(0.420, 0.631, 0.329)	(0.863, 0.163, 0.247)	(0.678, 0.388, 0.304)	(0.800, 0.245, 0.247)	(0.641, 0.438, 0.308)	(0.540, 0.510, 0.360)
BC5	(0.481, 0.601, 0.259)	(0.689, 0.372, 0.302)	(0.763, 0.261, 0.288)	(0.640, 0.445, 0.303)	(0.655, 0.429, 0.256)	(0.730, 0.314, 0.254)
BC6	(0.721, 0.330, 0.261)	(0.876, 0.141, 0.224)	(0.832, 0.180, 0.287)	(0.377, 0.662, 0.423)	(0.649, 0.429, 0.305)	(0.575, 0.482, 0.261)
BC7	(0.875, 0.145, 0.221)	(0.682, 0.338, 0.268)	(0.657, 0.429, 0.265)	(0.676, 0.375, 0.369)	(0.716, 0.310, 0.361)	(0.705, 0.350, 0.262)
BC8	(0.753, 0.317, 0.254)	(0.663, 0.421, 0.258)	(0.641, 0.393, 0.270)	(0.769, 0.299, 0.247)	(0.889, 0.120, 0.218)	(0.734, 0.304, 0.294)
BC9	(0.863, 0.163, 0.247)	(0.701, 0.353, 0.295)	(0.673, 0.392, 0.307)	(0.560, 0.571, 0.256)	(0.774, 0.282, 0.279)	(0.782, 0.258, 0.255)
BC10	(0.744, 0.300, 0.252)	(0.823, 0.210, 0.236)	(0.444, 0.592, 0.338)	(0.550, 0.485, 0.36)	(0.718, 0.328, 0.299)	(0.629, 0.476, 0.249)
BC11	(0.825, 0.207, 0.236)	(0.818, 0.202, 0.274)	(0.344, 0.697, 0.377)	(0.767, 0.308, 0.246)	(0.812, 0.221, 0.244)	(0.581, 0.470, 0.311)
BC12	(0.663, 0.421, 0.258)	(0.658, 0.445, 0.248)	(0.647, 0.439, 0.298)	(0.725, 0.312, 0.303)	(0.672, 0.382, 0.34)	(0.809, 0.228, 0.240)

**Table 6:** The removal effect matrix.

BC	OWC	PAB	ATE	SGP	TST	OTD
BC1	0.04	0.01	0.03	0.01	0	0.05
BC2	0.03	0.03	0.04	0.04	0.02	0
BC3	0.03	0.03	0.02	0	0	0.03
BC4	0	0.05	0.04	0.05	0.03	0.02
BC5	0	0.02	0.03	0.02	0.02	0.03
BC6	0.05	0.06	0.06	0	0.04	0.03
BC7	0.02	0	0	0	0	0
BC8	0.01	0	0	0.01	0.02	0.01
BC9	0.03	0.01	0.01	0	0.02	0.02
BC10	0.04	0.04	0	0.02	0.03	0.03
BC11	0.06	0.06	0	0.06	0.06	0.04
BC12	0	0	0	0.01	0	0.02

**Fig 3:**
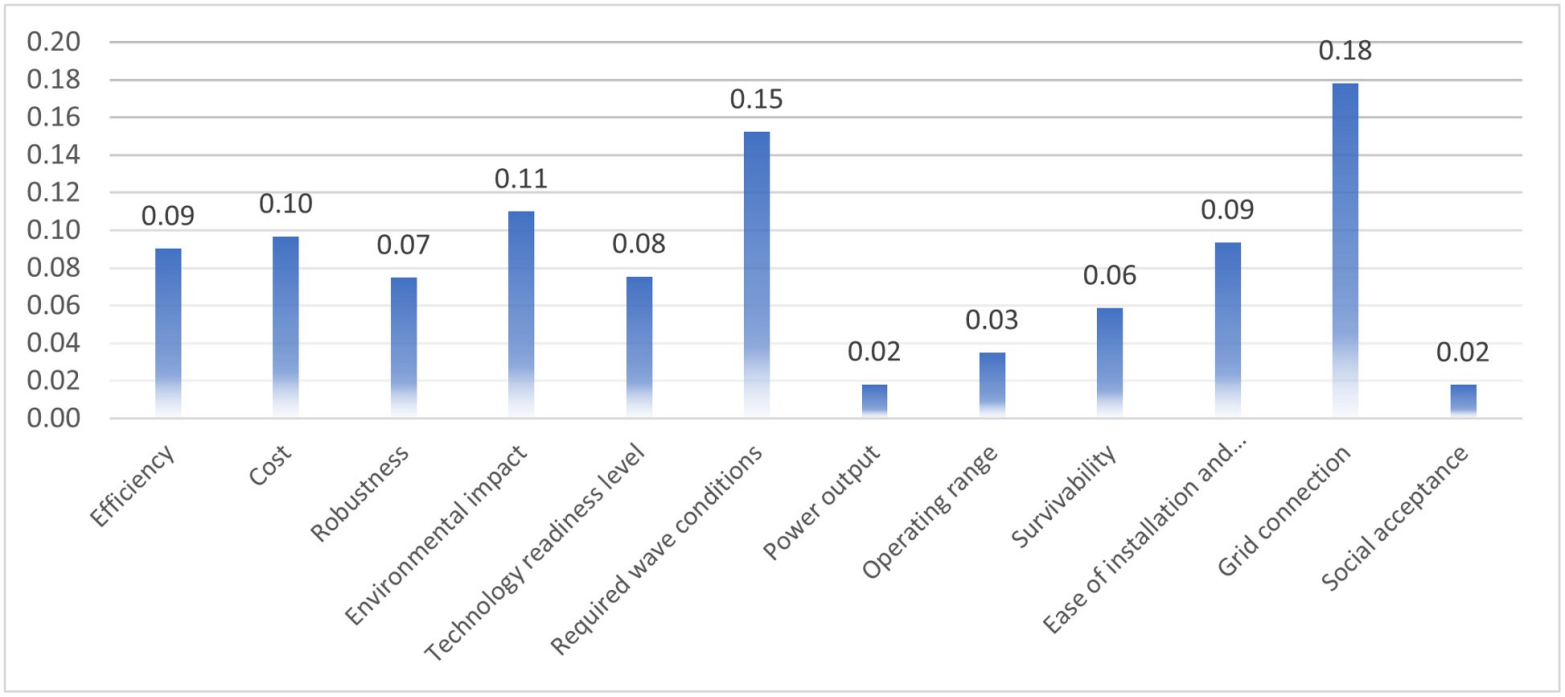
The objective weight of benchmarking criteria.

Then, the objective weight of the benchmarking criteria is determined according to Eq (26) and are illustrated in Fig 3. In the lights of weighting results, the highest weight assigned to Grid Connection, at 0.18, underscores the paramount importance of the ability of WECs to integrate with existing power grid infrastructures, a critical aspect for the practical deployment of these technologies. The Required Wave Conditions follow closely, with a weight of 0.15, reflecting the necessity for WEC technologies to operate efficiently across diverse marine environments, ensuring reliability and consistency in energy production. Cost, with a weight of 0.11, is emphasized as a major consideration, indicating that the economic viability of WEC technologies is crucial for their market penetration and scalability. Efficiency and Ease of Installation and Maintenance both garner a significant weight of 0.09, highlighting the balance between the effectiveness of energy conversion and the practical aspects of technology deployment and upkeep, which are key to the sustainable adoption and operation of WECs. Moderately weighted factors include Robustness and Environmental Impact, both at 0.07, suggesting these aspects are important but may not be as critical in differentiating between technologies as the top-weighted criteria. Survivability, at 0.06, also receives a moderate emphasis, indicating that the resilience of WECs to extreme marine conditions is an important factor in their overall evaluation. Power Output and Operating Range, each with a weight of 0.03, suggest that while these factors are integral to the function of WECs, they may be considered baseline expectations and not the primary drivers of technology selection. The Technology Readiness Level, at 0.08, is given a mid-range weight, which signifies a balanced emphasis on the maturity and development stage of the technology in the decision-making process. On the other hand, Social Acceptance is assigned the least weight, at 0.02, indicating that while societal factors are acknowledged, they may not be as influential in the technical and economic assessment phases of WEC technologies.

In the next stage, the aggregated SF benchmarking matrix is used to determine the Spherical weighted arithmetic mean (${\tilde{SWA}}_{i}$) and spherical weighted geometric mean (${\tilde{SWG}}_{i}$) based on Eqs (7) and (8). Then, their crisp values are obtained by Eq (15) and shown in Table 7. According to Eqs (28)–(30), the importance parameters of WEC technologies are defined as shown in Table 8. Ultimately, the final benchmark score is calculated according to Eq (31).

**Table 7:** The weighted sequences of WEC technologies.

WEC Technology	${\tilde{SWA}}_{i}$	${\tilde{SWG}}_{i}$	Crisp SWAM	Crisp SWGM
OWC	(0.742, 0.311, 0.262)	(0.691, 0.387, 0.268)	20.921	19.380
PAB	(0.789, 0.251, 0.262)	(0.753, 0.318, 0.260)	22.311	21.282
ATE	(0.668, 0.386, 0.318)	(0.596, 0.477, 0.330)	18.398	16.201
SGP	(0.662, 0.415, 0.301)	(0.597, 0.483, 0.330)	18.323	16.233
TST	(0.696, 0.363, 0.291)	(0.652, 0.428, 0.292)	19.380	18.093
OTD	(0.660, 0.404, 0.290)	(0.628, 0.440, 0.299)	18.323	17.312

**Table 8:** SF-CoCoSo importance parameters (*δ* = 0.5).

WEC Technology	Additive normalized importance ($\Phi_{i}^{a}$)	Relative importance ($\Phi_{i}^{b})$	Trade-off importance ($\Phi_{i}^{c})$	Final benchmark score (Φ_*i*_)
OWC	0.1782	2.3381	0.9245	1.8745
PAB	0.1928	2.5313	1.0000	2.0286
ATE	0.1530	2.0041	0.7937	1.6079
SGP	0.1528	2.0020	0.7927	1.6061
TST	0.1657	2.1745	0.8596	1.7432
OTD	0.1576	2.0686	0.8175	1.6580

### 4.2. Sensitivity analysis

**Fig 4:**
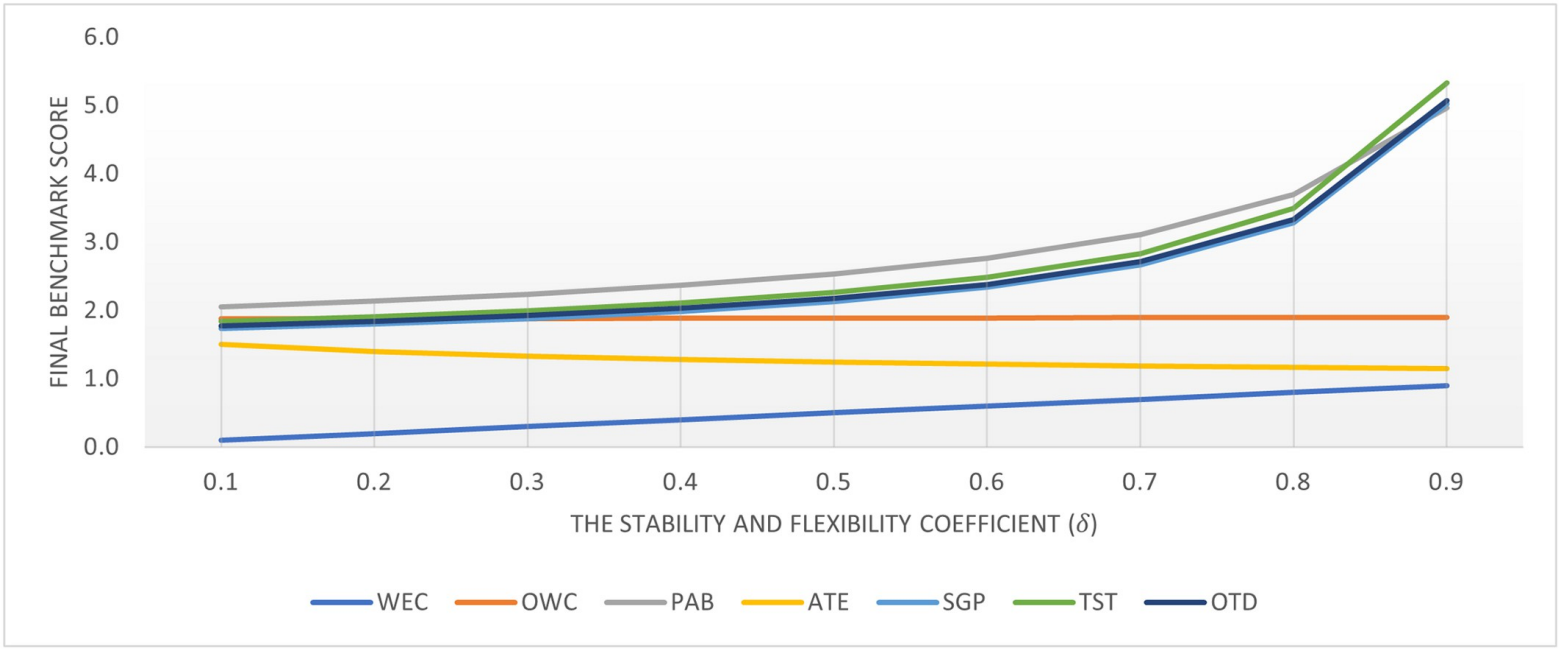
The final benchmarking score of WECs according to the stability and flexibility coefficients.

**Fig 5:**
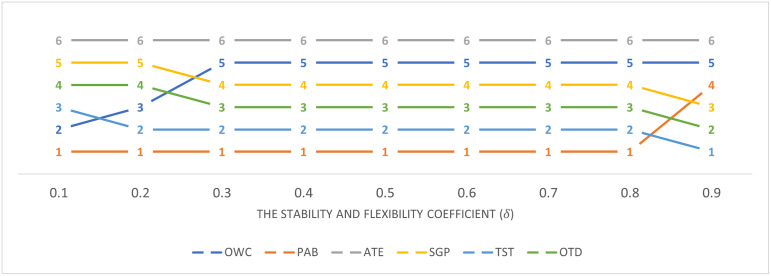
The ranking results of WECs according to the stability and flexibility coefficients.

In this section, the sensitivity analysis of the stability and flexibility coefficient (*δ*) with respect to the benchmarking results of WEC technologies reveals insightful trends and implications. The analysis spans a range of *δ* from 0.1 to 0.9, illustrating how variations in this coefficient impact the ranking and performance evaluation of WEC technologies as shown in Figs 4 and 5.

The OWC technology shows remarkable stability in its performance with scores consistently at 1.8 across a wide range of *δ* values, only increasing slightly to 1.9 at *δ* = 0.9. This consistency suggests that OWC technology’s performance is less sensitive to changes in the stability and flexibility coefficient, indicating a robust and reliable technology option. PAB and SGP technologies exhibit a high degree of sensitivity to changes in *δ*, with their scores increasing significantly as the coefficient rises. Specifically, PAB scores increase from 2.1 to 5.0, and SGP scores from 1.9 to 5.4 as *δ* increases from 0.1 to 0.9. This indicates that these technologies’ perceived effectiveness and suitability for wave energy conversion can vary greatly depending on the stability and flexibility requirements of the evaluation criteria. ATE shows a decreasing trend in performance as the *δ* value increases, with scores decreasing from 1.8 to 1.4. This trend suggests that ATE technology may become less favorable as more emphasis is placed on stability and flexibility in the decision-making process.

Notably, at higher *δ* values, PAB, SGP, TST, and OTD show marked increases in their scores, indicating a stronger preference for these technologies under conditions that highly value stability and flexibility. This shift emphasizes the importance of considering the operational environment and specific project needs when selecting WEC technologies. The dramatic increases in scores for PAB, SGP, TST, and OTD at high *δ* values (particularly at 0.9) suggest that the SF-CoCoSo method, under high stability and flexibility coefficients, may overestimate the advantages of certain technologies. This potential for overestimation underscores the necessity for careful consideration and calibration of the *δ* value to reflect realistic operational expectations and requirements.

## 5. Discussion

**Fig 6:**
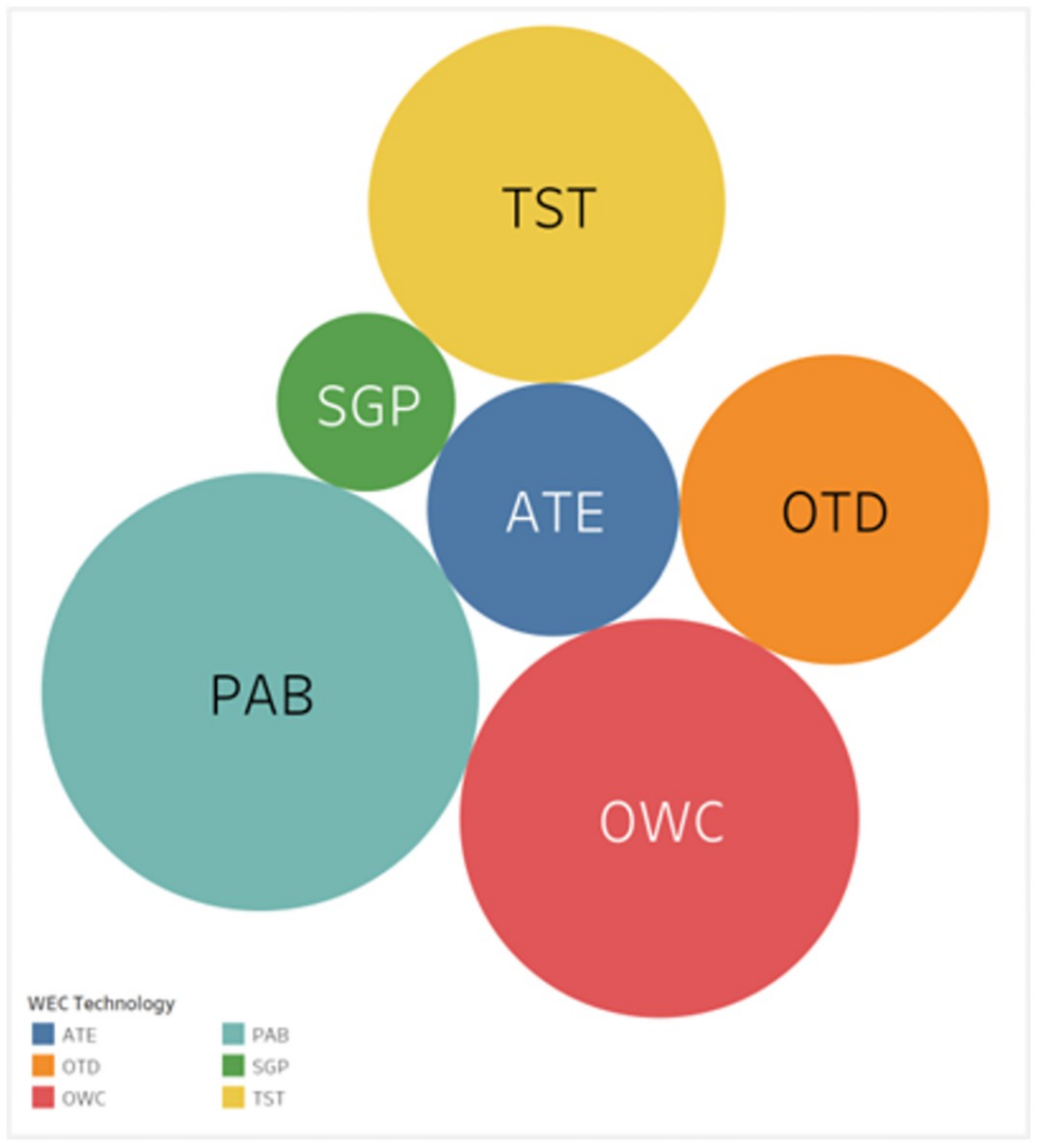
WEC technology final benchmarking score.

The benchmarking results for WEC technologies, as illustrated in Fig 6, offer a detailed perspective on the performance and suitability of each technology in relation to the criteria deemed important for successful deployment in the wave energy sector. At the forefront of these results is the PAB technology, which has achieved the highest score of 2.0286. This superior score suggests that PAB technology likely excels in several key areas such as efficiency, cost, grid compatibility, and possibly in its ability to operate across a range of wave conditions. The high score may also indicate that PAB technology aligns well with the current priorities and requirements of the industry, including aspects of environmental impact and social acceptance. The leading score of PAB technology points towards its potential as a frontrunner in the wave energy sector, setting a benchmark for others to aspire to. Following PAB, the OWC technology, with a score of 1.8745, and the TST technology, with a score of 1.7432, both show strong performances. These scores suggest that these technologies are likely to be competitive in the market, with strengths that may include robust design, high power output, and operational reliability. The slightly lower scores compared to PAB could indicate areas for improvement or could reflect strategic trade-offs in their design or operation that affect their overall benchmarking score.

The ATE and SGP technologies are closely ranked, with scores of 1.6079 and 1.6061, respectively. These similar scores may imply that both technologies share comparable capabilities or face similar challenges in meeting the benchmarking criteria. Their position towards the lower end of the performance spectrum could be attributed to factors such as higher costs, lower technology readiness levels, or perhaps less favorable environmental impacts. Nonetheless, these technologies might still offer specific advantages under certain conditions or for applications within the wave energy field. Lastly, the OTD technology, with a middle-lower score of 1.6580, suggests that while it may not excel across all criteria, it possesses a balanced suite of attributes that afford it a respectable place in the benchmarking evaluation. This score might be indicative of a technology with potential, one that could benefit from targeted improvements or could be well-suited to niche applications where its specific strengths are most valuable.

## 6. Managerial implications

This benchmarking study on WEC technologies using the MEREC and SF-CoCoSo methods offers valuable managerial insights that can significantly influence decision-making in the wave energy sector. For industry leaders and investors, the ranking of WEC technologies provides a strategic guide for directing investments toward the most efficient and reliable options. It allows for an informed allocation of research and development resources, particularly into high-potential areas that could enhance the performance and marketability of WECs. For policymakers, the findings can inform the creation of regulations and incentives that support the advancement and adoption of superior technologies. Manufacturers and developers of WEC technologies can utilize these insights to position their offerings more competitively, emphasizing the strengths identified through the benchmarking process in their marketing and communication strategies. Supply chain decisions can also be optimized based on the study’s outcomes. By aligning supply chain strategies with the production needs of the most promising WEC technologies, companies can achieve greater efficiency and cost-effectiveness. Furthermore, understanding the diverse risk profiles of each technology allows for the development of nuanced risk mitigation strategies, tailored to address specific technological vulnerabilities. The implications extend to investment diversification, suggesting that a balanced portfolio of WEC technologies might spread risk and increase resilience. This is particularly pertinent for companies looking to enter new markets or adapt existing technologies to meet local conditions and regulations. Sustainability considerations are also paramount. Managers can leverage the environmental impact data to steer their companies towards more sustainable practices and technologies, fulfilling corporate social responsibility objectives and enhancing the company’s reputation for environmental stewardship. Lastly, the social acceptance findings, albeit less weighted, are crucial for public relations and stakeholder engagement. They offer a framework for addressing public and governmental concerns, which is essential for obtaining project approvals and fostering community support. In essence, the managerial implications of this study are extensive, impacting investment, strategic planning, and operations. They provide a roadmap for enhancing competitive advantage and contribute to the sector’s progress towards sustainable and socially responsible energy solutions.

## 7. Conclusions

The study commenced with a focus on the burgeoning field of wave energy, recognizing the substantial untapped potential of ocean waves as a renewable energy source. Given the centrality of WECs in transforming wave power into electricity, the study aimed to evaluate and benchmark WEC technologies to determine the most efficient and viable solutions. To achieve this objective, the study employed an integrated approach, combining the MEREC and the SF-CoCoSo methods. MEREC was utilized to objectively weigh the various criteria crucial for evaluating WEC technologies, while SF-CoCoSo aided in aggregating and analyzing the complex decision-making data to derive a final evaluation score for each technology.

The study’s contributions are manifold. It provides a nuanced framework for benchmarking WEC technologies, thereby assisting stakeholders in making informed decisions. Additionally, the study advances the application of integrated MCDM approaches within the renewable energy sector, demonstrating the effectiveness of combining MEREC and SF-CoCoSo in a complex decision-making landscape. Our findings present a clear hierarchy of WEC technologies based on their performance across multiple criteria, including efficiency, cost, environmental impact, and grid connectivity. The study highlights the PAB technology as the front runner, with its superior overall performance, followed by the OWC and TST technologies as strong alternatives. Notably, it also emphasizes the importance of grid connection and adaptability to different wave conditions as critical factors in the benchmarking process.

Despite the valuable insights provided by this study, it recognizes a number of limitations that highlight areas for future exploration and development. The selected criteria for evaluating WEC technologies, while comprehensive, may not fully capture all the dimensions that influence their performance. This limitation opens up an avenue for future research to broaden the scope of evaluative criteria, incorporating emerging factors that could affect WEC technologies as advancements continue and new challenges arise in the field of renewable energy. Furthermore, the objectivity of the criteria weightings, despite being a strength of the current approach, might be subject to the shifting landscapes of the wave energy market and technological evolution. This suggests a need for adaptive methodologies that can dynamically adjust to the changing priorities and innovations within the sector. Future studies could focus on developing more flexible weighting mechanisms that respond to real-time market and technological data, thereby enhancing the relevance and timeliness of the benchmarking process. The assumption that the chosen MCDM methods adequately encapsulate the complexity inherent in the decision-making process for WEC technology assessment may not universally hold true. This indicates a promising research direction in exploring alternative MCDM methods that might offer different perspectives or handle specific aspects of the decision-making process more effectively. The exploration of these alternative methods could reveal new insights and possibly more efficient approaches to benchmarking WEC technologies. In the spirit of continuous improvement, this study serves as a pivotal step towards the systematic and rigorous benchmarking of WEC technologies. It emphasizes the importance of ongoing refinement of the assessment methodologies to align with technological advancements and market developments. Future research is thus encouraged not only to expand the criteria and explore alternative MCDM methods but also to implement strategies for validating the benchmarking process against real-world performance data. Such validation is crucial for ensuring the robustness and relevance of the findings, providing stakeholders with reliable and actionable insights. Moreover, there is an opportunity to integrate advancements in data analytics and artificial intelligence to enhance the benchmarking process. Future work could investigate the application of machine learning algorithms for predictive analysis and trend forecasting in the wave energy domain, offering a forward-looking component to the benchmarking process.

## Supporting information

S1 Appendix (DOCX)

S1 Dataset (DOCX)
