# Supplementary material for: Optimizing wave energy converter benchmarking with a fuzzy-based decision-making approach
Source: PLoS One. 2024 Jul 26;19(7):e0307894. doi: 10.1371/journal.pone.0307894 (PMC11280267; doi:10.1371/journal.pone.0307894)
Supplement: S1 Appendix — (DOCX) [file pone.0307894.s001.docx]

Appendix A

Table A1. The crisp benchmarking matrix

| BC | OWC | PAB | ATE | SGP | TST | OTD |
| --- | --- | --- | --- | --- | --- | --- |
| BC1 | 21.2 | 12.9 | 16 | 11.5 | 10.1 | 21.8 |
| BC2 | 18.5 | 21.2 | 20.4 | 22.2 | 16.9 | 12 |
| BC3 | 24.1 | 21.8 | 19.2 | 13.5 | 14.4 | 22.7 |
| BC4 | 11 | 24.6 | 18.8 | 22.7 | 17.7 | 14.3 |
| BC5 | 13.1 | 19.1 | 21.4 | 17.7 | 18.4 | 20.6 |
| BC6 | 20.3 | 25.1 | 23.4 | 9.14 | 17.9 | 15.9 |
| BC7 | 25.1 | 19.1 | 18.4 | 18.4 | 19.6 | 19.8 |
| BC8 | 21.3 | 18.6 | 17.9 | 21.8 | 25.5 | 20.5 |
| BC9 | 24.6 | 19.5 | 18.6 | 15.5 | 21.8 | 22.2 |
| BC10 | 21 | 23.5 | 11.6 | 14.7 | 20 | 17.6 |
| BC11 | 23.5 | 23.1 | 8.43 | 21.8 | 23.1 | 15.9 |
| BC12 | 18.6 | 18.5 | 17.9 | 20.2 | 18.4 | 23 |

Table A2. The normalized benchmarking matrix

| BC | OWC | PAB | ATE | SGP | TST | OTD |
| --- | --- | --- | --- | --- | --- | --- |
| BC1 | 0.48 | 0.78 | 0.63 | 0.88 | 1 | 0.46 |
| BC2 | 0.65 | 0.56 | 0.59 | 0.54 | 0.71 | 1 |
| BC3 | 0.56 | 0.62 | 0.7 | 1 | 0.94 | 0.59 |
| BC4 | 1 | 0.45 | 0.58 | 0.48 | 0.62 | 0.76 |
| BC5 | 1 | 0.69 | 0.61 | 0.74 | 0.71 | 0.64 |
| BC6 | 0.45 | 0.36 | 0.39 | 1 | 0.51 | 0.57 |
| BC7 | 0.73 | 0.96 | 1 | 1 | 0.94 | 0.93 |
| BC8 | 0.84 | 0.96 | 1 | 0.82 | 0.7 | 0.87 |
| BC9 | 0.63 | 0.8 | 0.83 | 1 | 0.71 | 0.7 |
| BC10 | 0.55 | 0.49 | 1 | 0.79 | 0.58 | 0.66 |
| BC11 | 0.36 | 0.36 | 1 | 0.39 | 0.36 | 0.53 |
| BC12 | 0.96 | 0.97 | 1 | 0.89 | 0.97 | 0.78 |
