# Supplementary material for: Optimizing wave energy converter benchmarking with a fuzzy-based decision-making approach
Source: PLoS One. 2024 Jul 26;19(7):e0307894. doi: 10.1371/journal.pone.0307894 (PMC11280267; doi:10.1371/journal.pone.0307894)
Supplement: S1 Dataset — (DOCX) [file pone.0307894.s002.docx]

**Minimal Data Set**

1. Expertise and weighting

| Expert | Linguistics term | SFN | Calculated expert weight |
| --- | --- | --- | --- |
| 1 | VH | (0.85, 0.15, 0.45) | 0.176 |
| 2 | VH | (0.85, 0.15, 0.45) | 0.176 |
| 3 | VH | (0.85, 0.15, 0.45) | 0.176 |
| 4 | H | (0.6, 0.2, 0.35) | 0.166 |
| 5 | H | (0.6, 0.2, 0.35) | 0.166 |
| 6 | M | (0.835, 0.25, 0.25) | 0.141 |

1. Linguistic assessment by experts

- Expert 1

| WECs | OWC | PAB | ATE | SGP | TST | OTD |
| --- | --- | --- | --- | --- | --- | --- |
| BC1 | L | SL | SL | SH | SL | L |
| BC2 | SL | SH | SL | L | SH | SH |
| BC3 | L | SL | SH | M | SL | L |
| BC4 | SL | VH | SH | SH | VH | SH |
| BC5 | L | SL | SH | VH | SL | SH |
| BC6 | VH | VH | SH | SL | SL | SH |
| BC7 | SH | L | L | VH | SH | SH |
| BC8 | L | SH | SH | VH | SH | VH |
| BC9 | VH | SH | M | L | M | SL |
| BC10 | SH | VH | SL | SL | M | L |
| BC11 | VH | M | M | VH | SL | L |
| BC12 | L | VH | M | M | SH | SH |

- Expert 2

| WECs | OWC | PAB | ATE | SGP | TST | OTD |
| --- | --- | --- | --- | --- | --- | --- |
| BC1 | SH | L | L | SL | SH | VH |
| BC2 | SH | VH | SH | SH | SH | SL |
| BC3 | SL | VH | VH | L | SH | SH |
| BC4 | SL | L | SL | VH | SL | M |
| BC5 | SL | VH | SH | SL | L | SH |
| BC6 | SH | SH | VH | SL | M | L |
| BC7 | VH | SH | SL | M | M | SL |
| BC8 | VH | L | SH | SL | VH | SH |
| BC9 | L | L | SL | SL | SL | SL |
| BC10 | SH | L | M | M | SH | SH |
| BC11 | SH | SH | SL | L | VH | L |
| BC12 | SH | L | L | SH | SL | SH |

- Expert 3

| WECs | OWC | PAB | ATE | SGP | TST | OTD |
| --- | --- | --- | --- | --- | --- | --- |
| BC1 | SH | SL | SL | L | SL | L |
| BC2 | SL | L | M | L | SL | M |
| BC3 | SL | L | M | SH | M | VH |
| BC4 | SL | M | VH | VH | SL | L |
| BC5 | SH | SH | VH | SL | VH | SH |
| BC6 | L | VH | SH | M | SH | L |
| BC7 | VH | SH | SL | L | VH | L |
| BC8 | SL | SL | SH | SL | SL | M |
| BC9 | M | M | SL | SL | VH | SH |
| BC10 | L | VH | SL | SH | SL | L |
| BC11 | VH | SH | SL | VH | L | SH |
| BC12 | VH | L | L | VH | M | L |

- Expert 4

| WECs | OWC | PAB | ATE | SGP | TST | OTD |
| --- | --- | --- | --- | --- | --- | --- |
| BC1 | VH | SH | SL | SL | L | VH |
| BC2 | SL | SH | VH | VH | M | L |
| BC3 | VH | L | L | SL | L | M |
| BC4 | SH | VH | L | SL | M | M |
| BC5 | SH | M | M | M | SL | L |
| BC6 | SL | VH | M | M | L | SH |
| BC7 | VH | SH | VH | M | M | VH |
| BC8 | SL | SL | L | L | VH | SH |
| BC9 | VH | SH | VH | L | L | VH |
| BC10 | L | SH | SL | L | L | SL |
| BC11 | L | SL | M | L | SH | SH |
| BC12 | SL | SL | SH | SH | L | VH |

- Expert 5

| WECs | OWC | PAB | ATE | SGP | TST | OTD |
| --- | --- | --- | --- | --- | --- | --- |
| BC1 | SH | L | SL | M | L | SL |
| BC2 | VH | SL | SH | SL | SH | M |
| BC3 | VH | VH | SL | L | SH | VH |
| BC4 | SL | VH | M | SL | L | L |
| BC5 | SL | L | SH | L | L | L |
| BC6 | SH | SL | M | M | L | SH |
| BC7 | L | SH | SH | M | M | SL |
| BC8 | SL | VH | SH | VH | VH | L |
| BC9 | VH | L | L | SL | VH | SL |
| BC10 | VH | SH | SH | SH | VH | L |
| BC11 | SH | VH | L | SL | SH | M |
| BC12 | SL | L | L | SL | M | L |

- Expert 6

| WECs | OWC | PAB | ATE | SGP | TST | OTD |
| --- | --- | --- | --- | --- | --- | --- |
| BC1 | SL | SH | VH | L | L | L |
| BC2 | L | SH | M | VH | M | SL |
| BC3 | VH | SL | SL | SH | SL | L |
| BC4 | M | SL | SL | L | SL | SH |
| BC5 | L | SL | L | L | SH | VH |
| BC6 | SL | SL | VH | L | VH | SL |
| BC7 | SL | SH | SL | SL | SL | SH |
| BC8 | VH | L | SL | L | SH | SL |
| BC9 | SL | VH | SH | VH | L | VH |
| BC10 | SH | L | SL | M | SH | VH |
| BC11 | L | VH | L | SL | VH | SH |
| BC12 | L | SH | VH | SL | VH | VH |

1. SFN assessment by experts

- Expert 1

| WECs | OWC | PAB | ATE | SGP | TST | OTD |
| --- | --- | --- | --- | --- | --- | --- |
| BC1 | (0.04, 0.96, 0.155) | (0.155, 0.845, 0.27) | (0.27, 0.73, 0.27) | (0.73, 0.27, 0.27) | (0.27, 0.73, 0.27) | (0.04, 0.96, 0.155) |
| BC2 | (0.155, 0.845, 0.27) | (0.73, 0.27, 0.27) | (0.155, 0.845, 0.27) | (0.04, 0.96, 0.155) | (0.73, 0.27, 0.27) | (0.73, 0.27, 0.27) |
| BC3 | (0.04, 0.96, 0.155) | (0.155, 0.845, 0.27) | (0.73, 0.27, 0.27) | (0.5, 0.5, 0.5) | (0.27, 0.73, 0.27) | (0.04, 0.96, 0.155) |
| BC4 | (0.155, 0.845, 0.27) | (0.96, 0.04, 0.155) | (0.73, 0.27, 0.27) | (0.73, 0.27, 0.27) | (0.96, 0.04, 0.155) | (0.73, 0.27, 0.27) |
| BC5 | (0.04, 0.96, 0.155) | (0.155, 0.845, 0.27) | (0.73, 0.27, 0.27) | (0.96, 0.04, 0.155) | (0.27, 0.73, 0.27) | (0.73, 0.27, 0.27) |
| BC6 | (0.96, 0.04, 0.155) | (0.96, 0.04, 0.155) | (0.73, 0.27, 0.27) | (0.155, 0.845, 0.27) | (0.155, 0.845, 0.27) | (0.73, 0.27, 0.27) |
| BC7 | (0.73, 0.27, 0.27) | (0.04, 0.96, 0.155) | (0.04, 0.96, 0.155) | (0.96, 0.04, 0.155) | (0.73, 0.27, 0.27) | (0.73, 0.27, 0.27) |
| BC8 | (0.04, 0.96, 0.155) | (0.73, 0.27, 0.27) | (0.73, 0.27, 0.27) | (0.96, 0.04, 0.155) | (0.73, 0.27, 0.27) | (0.96, 0.04, 0.155) |
| BC9 | (0.96, 0.04, 0.155) | (0.73, 0.27, 0.27) | (0.5, 0.5, 0.5) | (0.04, 0.96, 0.155) | (0.5, 0.5, 0.5) | (0.155, 0.845, 0.27) |
| BC10 | (0.73, 0.27, 0.27) | (0.96, 0.04, 0.155) | (0.27, 0.73, 0.27) | (0.27, 0.73, 0.27) | (0.5, 0.5, 0.5) | (0.04, 0.96, 0.155) |
| BC11 | (0.96, 0.04, 0.155) | (0.5, 0.5, 0.5) | (0.5, 0.5, 0.5) | (0.96, 0.04, 0.155) | (0.155, 0.845, 0.27) | (0.04, 0.96, 0.155) |
| BC12 | (0.04, 0.96, 0.155) | (0.96, 0.04, 0.155) | (0.5, 0.5, 0.5) | (0.5, 0.5, 0.5) | (0.73, 0.27, 0.27) | (0.73, 0.27, 0.27) |

- Expert 2

| WECs | OWC | PAB | ATE | SGP | TST | OTD |
| --- | --- | --- | --- | --- | --- | --- |
| BC1 | (0.73, 0.27, 0.27) | (0.04, 0.96, 0.155) | (0.04, 0.96, 0.155) | (0.27, 0.73, 0.27) | (0.73, 0.27, 0.27) | (0.96, 0.04, 0.155) |
| BC2 | (0.73, 0.27, 0.27) | (0.96, 0.04, 0.155) | (0.73, 0.27, 0.27) | (0.73, 0.27, 0.27) | (0.73, 0.27, 0.27) | (0.155, 0.845, 0.27) |
| BC3 | (0.27, 0.73, 0.27) | (0.96, 0.04, 0.155) | (0.96, 0.04, 0.155) | (0.04, 0.96, 0.155) | (0.73, 0.27, 0.27) | (0.73, 0.27, 0.27) |
| BC4 | (0.27, 0.73, 0.27) | (0.04, 0.96, 0.155) | (0.155, 0.845, 0.27) | (0.96, 0.04, 0.155) | (0.27, 0.73, 0.27) | (0.5, 0.5, 0.5) |
| BC5 | (0.155, 0.845, 0.27) | (0.96, 0.04, 0.155) | (0.73, 0.27, 0.27) | (0.27, 0.73, 0.27) | (0.04, 0.96, 0.155) | (0.73, 0.27, 0.27) |
| BC6 | (0.73, 0.27, 0.27) | (0.73, 0.27, 0.27) | (0.96, 0.04, 0.155) | (0.155, 0.845, 0.27) | (0.5, 0.5, 0.5) | (0.04, 0.96, 0.155) |
| BC7 | (0.96, 0.04, 0.155) | (0.73, 0.27, 0.27) | (0.155, 0.845, 0.27) | (0.5, 0.5, 0.5) | (0.5, 0.5, 0.5) | (0.27, 0.73, 0.27) |
| BC8 | (0.96, 0.04, 0.155) | (0.04, 0.96, 0.155) | (0.73, 0.27, 0.27) | (0.27, 0.73, 0.27) | (0.96, 0.04, 0.155) | (0.73, 0.27, 0.27) |
| BC9 | (0.04, 0.96, 0.155) | (0.04, 0.96, 0.155) | (0.155, 0.845, 0.27) | (0.155, 0.845, 0.27) | (0.27, 0.73, 0.27) | (0.27, 0.73, 0.27) |
| BC10 | (0.73, 0.27, 0.27) | (0.04, 0.96, 0.155) | (0.5, 0.5, 0.5) | (0.5, 0.5, 0.5) | (0.73, 0.27, 0.27) | (0.73, 0.27, 0.27) |
| BC11 | (0.73, 0.27, 0.27) | (0.73, 0.27, 0.27) | (0.27, 0.73, 0.27) | (0.04, 0.96, 0.155) | (0.96, 0.04, 0.155) | (0.04, 0.96, 0.155) |
| BC12 | (0.73, 0.27, 0.27) | (0.04, 0.96, 0.155) | (0.04, 0.96, 0.155) | (0.73, 0.27, 0.27) | (0.155, 0.845, 0.27) | (0.73, 0.27, 0.27) |

- Expert 3

| WECs | OWC | PAB | ATE | SGP | TST | OTD |
| --- | --- | --- | --- | --- | --- | --- |
| BC1 | (0.73, 0.27, 0.27) | (0.27, 0.73, 0.27) | (0.27, 0.73, 0.27) | (0.04, 0.96, 0.155) | (0.155, 0.845, 0.27) | (0.04, 0.96, 0.155) |
| BC2 | (0.155, 0.845, 0.27) | (0.04, 0.96, 0.155) | (0.5, 0.5, 0.5) | (0.04, 0.96, 0.155) | (0.155, 0.845, 0.27) | (0.5, 0.5, 0.5) |
| BC3 | (0.27, 0.73, 0.27) | (0.04, 0.96, 0.155) | (0.5, 0.5, 0.5) | (0.73, 0.27, 0.27) | (0.5, 0.5, 0.5) | (0.96, 0.04, 0.155) |
| BC4 | (0.155, 0.845, 0.27) | (0.5, 0.5, 0.5) | (0.96, 0.04, 0.155) | (0.96, 0.04, 0.155) | (0.155, 0.845, 0.27) | (0.04, 0.96, 0.155) |
| BC5 | (0.73, 0.27, 0.27) | (0.73, 0.27, 0.27) | (0.96, 0.04, 0.155) | (0.27, 0.73, 0.27) | (0.96, 0.04, 0.155) | (0.73, 0.27, 0.27) |
| BC6 | (0.04, 0.96, 0.155) | (0.96, 0.04, 0.155) | (0.73, 0.27, 0.27) | (0.5, 0.5, 0.5) | (0.73, 0.27, 0.27) | (0.04, 0.96, 0.155) |
| BC7 | (0.96, 0.04, 0.155) | (0.73, 0.27, 0.27) | (0.155, 0.845, 0.27) | (0.04, 0.96, 0.155) | (0.96, 0.04, 0.155) | (0.04, 0.96, 0.155) |
| BC8 | (0.155, 0.845, 0.27) | (0.27, 0.73, 0.27) | (0.73, 0.27, 0.27) | (0.155, 0.845, 0.27) | (0.27, 0.73, 0.27) | (0.5, 0.5, 0.5) |
| BC9 | (0.5, 0.5, 0.5) | (0.5, 0.5, 0.5) | (0.27, 0.73, 0.27) | (0.155, 0.845, 0.27) | (0.96, 0.04, 0.155) | (0.73, 0.27, 0.27) |
| BC10 | (0.04, 0.96, 0.155) | (0.96, 0.04, 0.155) | (0.27, 0.73, 0.27) | (0.73, 0.27, 0.27) | (0.155, 0.845, 0.27) | (0.04, 0.96, 0.155) |
| BC11 | (0.96, 0.04, 0.155) | (0.73, 0.27, 0.27) | (0.27, 0.73, 0.27) | (0.96, 0.04, 0.155) | (0.04, 0.96, 0.155) | (0.73, 0.27, 0.27) |
| BC12 | (0.96, 0.04, 0.155) | (0.04, 0.96, 0.155) | (0.04, 0.96, 0.155) | (0.96, 0.04, 0.155) | (0.5, 0.5, 0.5) | (0.04, 0.96, 0.155) |

- Expert 4

| WECs | OWC | PAB | ATE | SGP | TST | OTD |
| --- | --- | --- | --- | --- | --- | --- |
| BC1 | (0.96, 0.04, 0.155) | (0.73, 0.27, 0.27) | (0.155, 0.845, 0.27) | (0.27, 0.73, 0.27) | (0.04, 0.96, 0.155) | (0.96, 0.04, 0.155) |
| BC2 | (0.155, 0.845, 0.27) | (0.73, 0.27, 0.27) | (0.96, 0.04, 0.155) | (0.96, 0.04, 0.155) | (0.5, 0.5, 0.5) | (0.04, 0.96, 0.155) |
| BC3 | (0.96, 0.04, 0.155) | (0.04, 0.96, 0.155) | (0.04, 0.96, 0.155) | (0.155, 0.845, 0.27) | (0.04, 0.96, 0.155) | (0.5, 0.5, 0.5) |
| BC4 | (0.73, 0.27, 0.27) | (0.96, 0.04, 0.155) | (0.04, 0.96, 0.155) | (0.155, 0.845, 0.27) | (0.5, 0.5, 0.5) | (0.5, 0.5, 0.5) |
| BC5 | (0.73, 0.27, 0.27) | (0.5, 0.5, 0.5) | (0.5, 0.5, 0.5) | (0.5, 0.5, 0.5) | (0.27, 0.73, 0.27) | (0.04, 0.96, 0.155) |
| BC6 | (0.27, 0.73, 0.27) | (0.96, 0.04, 0.155) | (0.5, 0.5, 0.5) | (0.5, 0.5, 0.5) | (0.04, 0.96, 0.155) | (0.73, 0.27, 0.27) |
| BC7 | (0.96, 0.04, 0.155) | (0.73, 0.27, 0.27) | (0.96, 0.04, 0.155) | (0.5, 0.5, 0.5) | (0.5, 0.5, 0.5) | (0.96, 0.04, 0.155) |
| BC8 | (0.27, 0.73, 0.27) | (0.155, 0.845, 0.27) | (0.04, 0.96, 0.155) | (0.04, 0.96, 0.155) | (0.96, 0.04, 0.155) | (0.73, 0.27, 0.27) |
| BC9 | (0.96, 0.04, 0.155) | (0.73, 0.27, 0.27) | (0.96, 0.04, 0.155) | (0.04, 0.96, 0.155) | (0.04, 0.96, 0.155) | (0.96, 0.04, 0.155) |
| BC10 | (0.04, 0.96, 0.155) | (0.73, 0.27, 0.27) | (0.155, 0.845, 0.27) | (0.04, 0.96, 0.155) | (0.04, 0.96, 0.155) | (0.155, 0.845, 0.27) |
| BC11 | (0.04, 0.96, 0.155) | (0.155, 0.845, 0.27) | (0.5, 0.5, 0.5) | (0.04, 0.96, 0.155) | (0.73, 0.27, 0.27) | (0.73, 0.27, 0.27) |
| BC12 | (0.155, 0.845, 0.27) | (0.155, 0.845, 0.27) | (0.73, 0.27, 0.27) | (0.73, 0.27, 0.27) | (0.04, 0.96, 0.155) | (0.96, 0.04, 0.155) |

- Expert 5

| WECs | OWC | PAB | ATE | SGP | TST | OTD |
| --- | --- | --- | --- | --- | --- | --- |
| BC1 | (0.73, 0.27, 0.27) | (0.04, 0.96, 0.155) | (0.27, 0.73, 0.27) | (0.5, 0.5, 0.5) | (0.04, 0.96, 0.155) | (0.27, 0.73, 0.27) |
| BC2 | (0.96, 0.04, 0.155) | (0.155, 0.845, 0.27) | (0.73, 0.27, 0.27) | (0.27, 0.73, 0.27) | (0.73, 0.27, 0.27) | (0.5, 0.5, 0.5) |
| BC3 | (0.96, 0.04, 0.155) | (0.96, 0.04, 0.155) | (0.155, 0.845, 0.27) | (0.04, 0.96, 0.155) | (0.73, 0.27, 0.27) | (0.96, 0.04, 0.155) |
| BC4 | (0.155, 0.845, 0.27) | (0.96, 0.04, 0.155) | (0.5, 0.5, 0.5) | (0.155, 0.845, 0.27) | (0.04, 0.96, 0.155) | (0.04, 0.96, 0.155) |
| BC5 | (0.155, 0.845, 0.27) | (0.04, 0.96, 0.155) | (0.73, 0.27, 0.27) | (0.04, 0.96, 0.155) | (0.04, 0.96, 0.155) | (0.04, 0.96, 0.155) |
| BC6 | (0.73, 0.27, 0.27) | (0.27, 0.73, 0.27) | (0.5, 0.5, 0.5) | (0.5, 0.5, 0.5) | (0.04, 0.96, 0.155) | (0.73, 0.27, 0.27) |
| BC7 | (0.04, 0.96, 0.155) | (0.73, 0.27, 0.27) | (0.73, 0.27, 0.27) | (0.5, 0.5, 0.5) | (0.5, 0.5, 0.5) | (0.155, 0.845, 0.27) |
| BC8 | (0.155, 0.845, 0.27) | (0.96, 0.04, 0.155) | (0.73, 0.27, 0.27) | (0.96, 0.04, 0.155) | (0.96, 0.04, 0.155) | (0.04, 0.96, 0.155) |
| BC9 | (0.96, 0.04, 0.155) | (0.04, 0.96, 0.155) | (0.04, 0.96, 0.155) | (0.155, 0.845, 0.27) | (0.96, 0.04, 0.155) | (0.27, 0.73, 0.27) |
| BC10 | (0.96, 0.04, 0.155) | (0.73, 0.27, 0.27) | (0.73, 0.27, 0.27) | (0.73, 0.27, 0.27) | (0.96, 0.04, 0.155) | (0.04, 0.96, 0.155) |
| BC11 | (0.73, 0.27, 0.27) | (0.96, 0.04, 0.155) | (0.04, 0.96, 0.155) | (0.155, 0.845, 0.27) | (0.73, 0.27, 0.27) | (0.5, 0.5, 0.5) |
| BC12 | (0.27, 0.73, 0.27) | (0.04, 0.96, 0.155) | (0.04, 0.96, 0.155) | (0.155, 0.845, 0.27) | (0.5, 0.5, 0.5) | (0.04, 0.96, 0.155) |

- Expert 6

| WECs | OWC | PAB | ATE | SGP | TST | OTD |
| --- | --- | --- | --- | --- | --- | --- |
| BC1 | (0.27, 0.73, 0.27) | (0.73, 0.27, 0.27) | (0.96, 0.04, 0.155) | (0.04, 0.96, 0.155) | (0.04, 0.96, 0.155) | (0.04, 0.96, 0.155) |
| BC2 | (0.04, 0.96, 0.155) | (0.73, 0.27, 0.27) | (0.5, 0.5, 0.5) | (0.96, 0.04, 0.155) | (0.5, 0.5, 0.5) | (0.155, 0.845, 0.27) |
| BC3 | (0.96, 0.04, 0.155) | (0.155, 0.845, 0.27) | (0.27, 0.73, 0.27) | (0.73, 0.27, 0.27) | (0.27, 0.73, 0.27) | (0.04, 0.96, 0.155) |
| BC4 | (0.5, 0.5, 0.5) | (0.27, 0.73, 0.27) | (0.155, 0.845, 0.27) | (0.04, 0.96, 0.155) | (0.27, 0.73, 0.27) | (0.73, 0.27, 0.27) |
| BC5 | (0.04, 0.96, 0.155) | (0.27, 0.73, 0.27) | (0.04, 0.96, 0.155) | (0.04, 0.96, 0.155) | (0.73, 0.27, 0.27) | (0.96, 0.04, 0.155) |
| BC6 | (0.155, 0.845, 0.27) | (0.155, 0.845, 0.27) | (0.96, 0.04, 0.155) | (0.04, 0.96, 0.155) | (0.96, 0.04, 0.155) | (0.27, 0.73, 0.27) |
| BC7 | (0.27, 0.73, 0.27) | (0.73, 0.27, 0.27) | (0.155, 0.845, 0.27) | (0.27, 0.73, 0.27) | (0.27, 0.73, 0.27) | (0.73, 0.27, 0.27) |
| BC8 | (0.96, 0.04, 0.155) | (0.04, 0.96, 0.155) | (0.155, 0.845, 0.27) | (0.04, 0.96, 0.155) | (0.73, 0.27, 0.27) | (0.27, 0.73, 0.27) |
| BC9 | (0.27, 0.73, 0.27) | (0.96, 0.04, 0.155) | (0.73, 0.27, 0.27) | (0.96, 0.04, 0.155) | (0.04, 0.96, 0.155) | (0.96, 0.04, 0.155) |
| BC10 | (0.73, 0.27, 0.27) | (0.04, 0.96, 0.155) | (0.27, 0.73, 0.27) | (0.5, 0.5, 0.5) | (0.73, 0.27, 0.27) | (0.96, 0.04, 0.155) |
| BC11 | (0.04, 0.96, 0.155) | (0.96, 0.04, 0.155) | (0.04, 0.96, 0.155) | (0.155, 0.845, 0.27) | (0.96, 0.04, 0.155) | (0.73, 0.27, 0.27) |
| BC12 | (0.04, 0.96, 0.155) | (0.73, 0.27, 0.27) | (0.96, 0.04, 0.155) | (0.155, 0.845, 0.27) | (0.96, 0.04, 0.155) | (0.96, 0.04, 0.155) |

1. The aggregated SF benchmarking matrix

| **BC** | **OWC** | **PAB** | **ATE** | **SGP** | **TST** | **OTD** |
| --- | --- | --- | --- | --- | --- | --- |
| BC1 | (0.750, 0.284, 0.257) | (0.473, 0.605, 0.256) | (0.578, 0.518, 0.265) | (0.435, 0.625, 0.319) | (0.378, 0.714, 0.240) | (0.768, 0.306, 0.240) |
| BC2 | (0.661, 0.422, 0.266) | (0.751, 0.287, 0.258) | (0.736, 0.291, 0.323) | (0.780, 0.273, 0.244) | (0.623, 0.395, 0.353) | (0.462, 0.591, 0.373) |
| BC3 | (0.843, 0.192, 0.233) | (0.767, 0.308, 0.246) | (0.692, 0.367, 0.302) | (0.503, 0.565, 0.319) | (0.534, 0.509, 0.322) | (0.803, 0.238, 0.269) |
| BC4 | (0.420, 0.631, 0.329) | (0.863, 0.163, 0.247) | (0.678, 0.388, 0.304) | (0.800, 0.245, 0.247) | (0.641, 0.438, 0.308) | (0.540, 0.510, 0.360) |
| BC5 | (0.481, 0.601, 0.259) | (0.689, 0.372, 0.302) | (0.763, 0.261, 0.288) | (0.640, 0.445, 0.303) | (0.655, 0.429, 0.256) | (0.730, 0.314, 0.254) |
| BC6 | (0.721, 0.330, 0.261) | (0.876, 0.141, 0.224) | (0.832, 0.180, 0.287) | (0.377, 0.662, 0.423) | (0.649, 0.429, 0.305) | (0.575, 0.482, 0.261) |
| BC7 | (0.875, 0.145, 0.221) | (0.682, 0.338, 0.268) | (0.657, 0.429, 0.265) | (0.676, 0.375, 0.369) | (0.716, 0.310, 0.361) | (0.705, 0.350, 0.262) |
| BC8 | (0.753, 0.317, 0.254) | (0.663, 0.421, 0.258) | (0.641, 0.393, 0.270) | (0.769, 0.299, 0.247) | (0.889, 0.120, 0.218) | (0.734, 0.304, 0.294) |
| BC9 | (0.863, 0.163, 0.247) | (0.701, 0.353, 0.295) | (0.673, 0.392, 0.307) | (0.560, 0.571, 0.256) | (0.774, 0.282, 0.279) | (0.782, 0.258, 0.255) |
| BC10 | (0.744, 0.300, 0.252) | (0.823, 0.210, 0.236) | (0.444, 0.592, 0.338) | (0.550, 0.485, 0.36) | (0.718, 0.328, 0.299) | (0.629, 0.476, 0.249) |
| BC11 | (0.825, 0.207, 0.236) | (0.818, 0.202, 0.274) | (0.344, 0.697, 0.377) | (0.767, 0.308, 0.246) | (0.812, 0.221, 0.244) | (0.581, 0.470, 0.311) |
| BC12 | (0.663, 0.421, 0.258) | (0.658, 0.445, 0.248) | (0.647, 0.439, 0.298) | (0.725, 0.312, 0.303) | (0.672, 0.382, 0.34) | (0.809, 0.228, 0.240) |

1. The defuzzied SF benchmarking matrix

| WECs | BC1 | BC2 | BC3 | BC4 | BC5 | BC6 | BC7 | BC8 | BC9 | BC10 | BC11 | BC12 |
| --- | --- | --- | --- | --- | --- | --- | --- | --- | --- | --- | --- | --- |
| OWC | 21.2 | 18.5 | 24.1 | 11 | 13.1 | 20.3 | 25.1 | 21.3 | 24.6 | 21 | 23.5 | 18.6 |
| PAB | 12.9 | 21.2 | 21.8 | 24.6 | 19.1 | 25.1 | 19.1 | 18.6 | 19.5 | 23.5 | 23.1 | 18.5 |
| ATE | 16 | 20.4 | 19.2 | 18.8 | 21.4 | 23.4 | 18.4 | 17.9 | 18.6 | 11.6 | 8.43 | 17.9 |
| SGP | 11.5 | 22.2 | 13.5 | 22.7 | 17.7 | 9.14 | 18.4 | 21.8 | 15.5 | 14.7 | 21.8 | 20.2 |
| TST | 10.1 | 16.9 | 14.4 | 17.7 | 18.4 | 17.9 | 19.6 | 25.5 | 21.8 | 20 | 23.1 | 18.4 |
| OTD | 21.8 | 12 | 22.7 | 14.3 | 20.6 | 15.9 | 19.8 | 20.5 | 22.2 | 17.6 | 15.9 | 23 |

1. The removal effect matrix

| BC | OWC | PAB | ATE | SGP | TST | OTD | **Sum** | **Weight** |
| --- | --- | --- | --- | --- | --- | --- | --- | --- |
| BC1 | 0.04 | 0.01 | 0.03 | 0.01 | 0 | 0.05 | **0.15** | **0.09** |
| BC2 | 0.03 | 0.03 | 0.04 | 0.04 | 0.02 | 0 | **0.16** | **0.1** |
| BC3 | 0.03 | 0.03 | 0.02 | 0 | 0 | 0.03 | **0.12** | **0.07** |
| BC4 | 0 | 0.05 | 0.04 | 0.05 | 0.03 | 0.02 | **0.18** | **0.11** |
| BC5 | 0 | 0.02 | 0.03 | 0.02 | 0.02 | 0.03 | **0.12** | **0.08** |
| BC6 | 0.05 | 0.06 | 0.06 | 0 | 0.04 | 0.03 | **0.25** | **0.15** |
| BC7 | 0.02 | 0 | 0 | 0 | 0 | 0 | **0.03** | **0.02** |
| BC8 | 0.01 | 0 | 0 | 0.01 | 0.02 | 0.01 | **0.06** | **0.03** |
| BC9 | 0.03 | 0.01 | 0.01 | 0 | 0.02 | 0.02 | **0.09** | **0.06** |
| BC10 | 0.04 | 0.04 | 0 | 0.02 | 0.03 | 0.03 | **0.15** | **0.09** |
| BC11 | 0.06 | 0.06 | 0 | 0.06 | 0.06 | 0.04 | **0.29** | **0.18** |
| BC12 | 0 | 0 | 0 | 0.01 | 0 | 0.02 | **0.03** | **0.02** |

1. The weighted sequences of WEC technologies

| WEC Technology | $\tilde{SWA}_{i}$ | $\tilde{SWG}_{i}$ | Crisp SWAM | Crisp SWGM |
| --- | --- | --- | --- | --- |
| OWC | (0.742, 0.311, 0.262) | (0.691, 0.387, 0.268) | 20.921 | 19.380 |
| PAB | (0.789, 0.251, 0.262) | (0.753, 0.318, 0.260) | 22.311 | 21.282 |
| ATE | (0.668, 0.386, 0.318) | (0.596, 0.477, 0.330) | 18.398 | 16.201 |
| SGP | (0.662, 0.415, 0.301) | (0.597, 0.483, 0.330) | 18.323 | 16.233 |
| TST | (0.696, 0.363, 0.291) | (0.652, 0.428, 0.292) | 19.380 | 18.093 |
| OTD | (0.660, 0.404, 0.290) | (0.628, 0.440, 0.299) | 18.323 | 17.312 |

1. SF-CoCoSo importance parameters ($\boldsymbol{\delta}=\mathbf{0}.\mathbf{5})$

| WEC Technology | Additive normalized importance  ($\Phi_{i}^{a}$) | Relative importance  ($\Phi_{i}^{b})$ | Trade‐off importance  ($\Phi_{i}^{c})$ | Final benchmark score  $(\Phi_{i})$ |
| --- | --- | --- | --- | --- |
| OWC | 0.1782 | 2.3381 | 0.9245 | 1.8745 |
| PAB | 0.1928 | 2.5313 | 1.0000 | 2.0286 |
| ATE | 0.1530 | 2.0041 | 0.7937 | 1.6079 |
| SGP | 0.1528 | 2.0020 | 0.7927 | 1.6061 |
| TST | 0.1657 | 2.1745 | 0.8596 | 1.7432 |
| OTD | 0.1576 | 2.0686 | 0.8175 | 1.6580 |

1. Sensitivity analysis

| WEC | Stability and flexibility coefficient | | | | | | | | |
| --- | --- | --- | --- | --- | --- | --- | --- | --- | --- |
|  | 0.1 | 0.2 | 0.3 | 0.4 | 0.5 | 0.6 | 0.7 | 0.8 | 0.9 |
| OWC | 1.9 | 1.9 | 1.9 | 1.9 | 1.9 | 1.9 | 1.9 | 1.9 | 1.9 |
| PAB | 2.1 | 2.1 | 2.2 | 2.4 | 2.5 | 2.8 | 3.1 | 3.7 | 5.0 |
| ATE | 1.5 | 1.4 | 1.3 | 1.3 | 1.2 | 1.2 | 1.2 | 1.2 | 1.1 |
| SGP | 1.7 | 1.8 | 1.9 | 2.0 | 2.1 | 2.3 | 2.7 | 3.3 | 5.0 |
| TST | 1.8 | 1.9 | 2.0 | 2.1 | 2.3 | 2.5 | 2.8 | 3.5 | 5.3 |
| OTD | 1.8 | 1.8 | 1.9 | 2.0 | 2.2 | 2.4 | 2.7 | 3.3 | 5.1 |
